# Molecular mechanisms of tannin accumulation in Rhus galls and genes involved in plant-insect interactions

**DOI:** 10.1038/s41598-018-28153-y

**Published:** 2018-06-29

**Authors:** Hang Chen, Juan Liu, Kai Cui, Qin Lu, Chao Wang, Haixia Wu, Zixiang Yang, Weifeng Ding, Shuxia Shao, Haiying Wang, Xiaofei Ling, Kirst King-Jones, Xiaoming Chen

**Affiliations:** 1 Research Institute of Resource Insects, Chinese Academy of Forestry, Kunming, China; 2 The Key Laboratory of Cultivating and Utilization of Resources Insects, State Forestry Administration, Kunming, China; 3 Southwest Forestry University, Kunming, 650224 Yunnan China; 4 Department of Biological Sciences, Biological Sciences Building, University of Alberta, Edmonton, Canada

## Abstract

For galling aphids and their hosts, tannins are crucial for plant-insect interactions and for protecting the host plant from herbivory. Due to their peculiar chemical characteristics, tannins from plant galls have been used for medical and chemical purposes for more than 2000 years. In this study, hydrolyzable tannin concentrations in galls increased from gall initiation (38.34% on June 21) to maturation (74.79% on August 8), then decreased gradually thereafter (58.83% on October 12). We identified a total of 81 genes (named as *GTS1-81*) with putative roles in gallotannin biosynthesis and 22 genes (*TS1-22*) in condensed tannin biosynthesis. We determined the expression profiles of these genes by real-time PCR over the course of gall development. Multiple genes encoding 1-beta-D-glucosyl transferases were identified, which may play a vital role in gallotannin accumulation in plant galls. This study is the first attempt to examine the molecular basis for the regulation of tannin accumulation in insect gallnuts. The differentially expressed genes we identified may play important roles in both tannin biosynthesis and plant-insect interactions.

## Introduction

Plants and insects have co-existed for more than 350 million years^1^, and in their interactions both have evolved strategies to overcome each other’s defense systems^2,3^. Many insect species induce the formation of galls on various plant tissues. The aphid *Schlechtendalia chinensis* (Bell) is a tiny insect of the Pemphigidae family (Hemiptera: Aphidoidea). It feeds on the adaxial surface of winged rachides and induces the formation of large, single-chambered galls, called horned galls, on *R. chinensis*^4^. The galls have been used for medical and chemical applications as a source of tannic, gallic, and pyrogallic acids for more than 2,000 years. It has been reported that there are at least 15,000 tons of tannin demands in different industrial fields including leatherworking, textile printing and mineral separation each year around the world (Fig. S1). And the whole value of industry related to tannins has added up to 30 billion dollars annually^5–8^.

Tannins are astringent, polyphenolic compounds that bind to and precipitate proteins and various other organic compounds including amino acids and alkaloids. Tannins occur widely in various plant tissues, where they play crucial roles in protecting plants from herbivory, and in regulating plant growth^9^. Most tannins have molecular weights from 500 to over 3,000 (gallic acid esters) Da, but some can reach up to 20,000 (proanthocyanidins) (Fig. S2)^10,11^. Plant tannins are divided into hydrolyzable tannins and flavonoid-derived, condensed tannins, which are also called proanthocyanidins. Condensed tannins are produced through the shikimate pathway leading to the production of anthocyanins, a pathway that has been investigated intensively at biochemical and genetic levels (Fig. S2)^12–14^. The current understanding is that anthocyanins and the other flavonoid classes are derived from earlier intermediates in the anthocyanin pathway^15–17^.

Hydrolysable tannins are derivatives of gallic acids. The simplest hydrolysable tannins, the gallotannins, are simple polygalloyl esters of glucose^18^. The prototypical gallotannin is pentagalloyl glucose, and has five identical ester linkages that involve aliphatic hydroxyl groups of the core sugar (Fig. S2). The biosynthetic pathways of hydrolyzable tannins include the biosynthesis of three intermediates, pentagalloylglucoses, gallotannins, and ellagitannins^19^.

**Figure 1:**
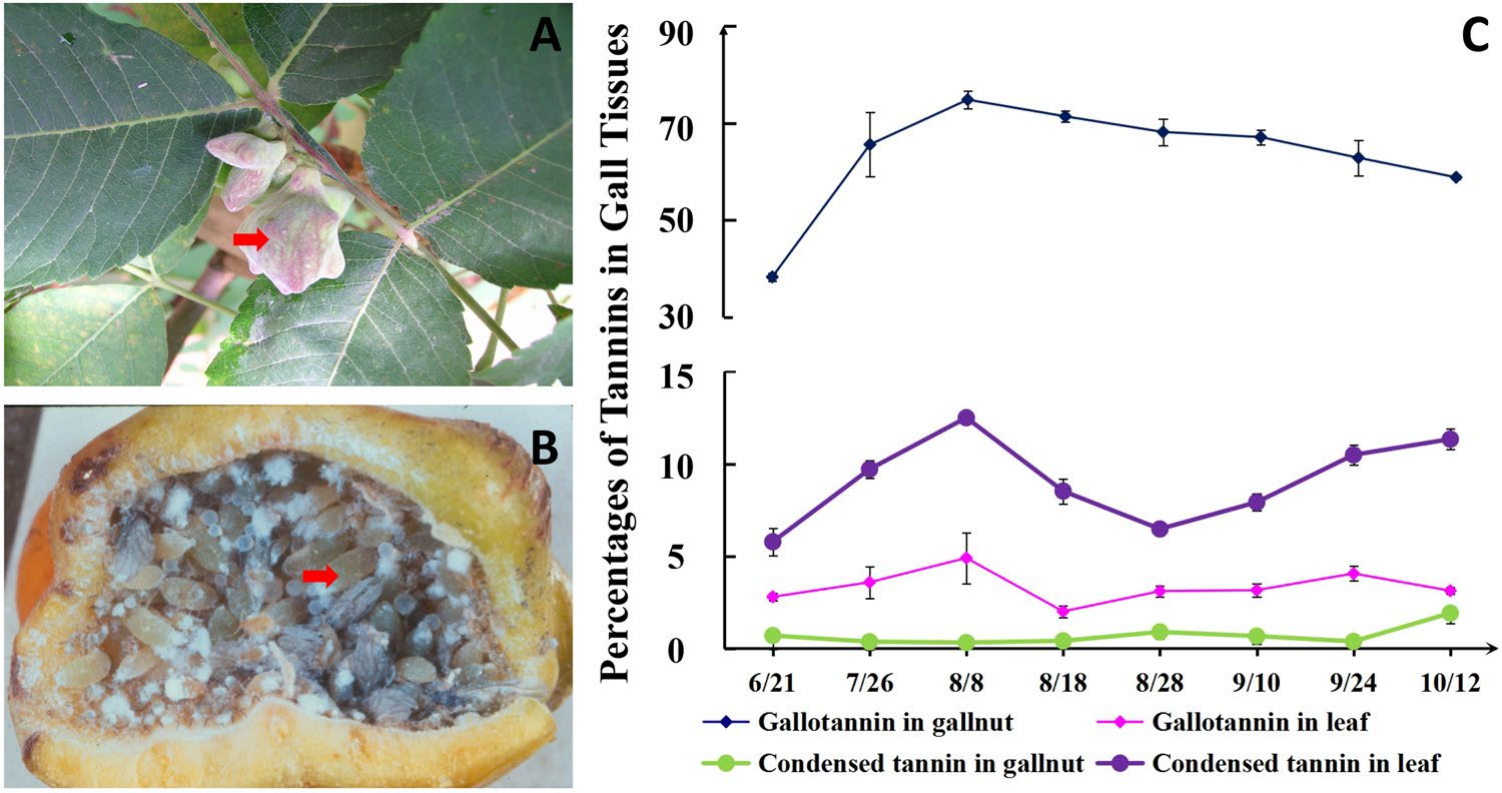
Changes in tannin concentrations in galls and leaves over the course of gallnut growth. (**A**) The fundatrix feeds on the winged rachides and initiates gall formation, the arrow pointing the location of early stage of gall on the leaf; (**B**) hundreds of aphids inside horned gall stimulating the gall growing constantly, the arrow pointing the individual aphid; (**C**) the change trend of two kinds of tannin contents in gall and in leaves with the growth of gallnut. The horizontal coordinates are the dry weight percentage of tannins; the vertical coordinates are different developmental stage of gallnut from June 21 to October 12.

*Schlechtendalia chinensis* is an insect with a complex life cycle that includes the development of gall-dwelling colonies on its host *R. chinensis*. In early spring in China, sexuparae migrate from the moss to the trunk of *R. chinensis* tree to produce sexually reproductive females and males that subsequently mate to produce fundatrices. A fundatrix feeds on the winged rachides of *R. chinensis* and then initiates gall formation (Fig. 1A,B)^20,21^. After a horned gall matures and dehisces in autumn, the alate fundatrigeniae migrate from the gall to the moss, its over-wintering host^22^.

Although the biological characteristics of *R. chinensis* and its herbivore *S. chinensis* have been examined by different labs over the years^23,24^, at present, there are no genomic resources available for *R. chinensis*. A better understanding of the molecular underpinnings for tannin biosynthesis would potentially benefit the tannin industry, which is currently hampered by the lack of information available on transcriptional, proteomic, and genetic analyses of *R. chinensis*. Next generation sequencing technologies have proven to be a rapid and cost-effective means for analyzing the genome and transcriptome of non-model species^25–27^. Our long-term goal is to identify genes with functions in the biosynthesis of tannins and to modify these genes genetically to increase the efficiency of tannin production. The specific objective of this study was to generate a transcriptome that provides a foundation for future studies leading to a better understanding ofwhy tannin accumulation occurs so rapidly in galls growing on *R. chinensis* and the co-evolution trends between insects and their host plants.

## Results

### Accumulation of tannins in horned galls and leaves

To determine the best time for sample collection for transcriptomic analyses, we measured gallotannin concentrations in galls and leaves throughout an entire season (June through October). Gallotannin concentrations in galls were nearly doubled in the first six weeks, from 38.34% on June 21 to 74.79% of dry gallnut weight on August 8. After that, gallotannin concentrations gradually decreased to 58.83% of dry gallnut weight over the next ten weeks (Fig. 1C, Table S1). The concentrations of gallotannin in leaf samples were considerably lower compared to those in gallnuts. Still, there was a significant increase in gallotanninin leaves during the process of gall initiation to maturation. On June 21, at the beginning of gallnut formation, the gallotannin abundance in leaves was only 2.79% of dry weight (Table S2), which was then nearly doubled to 4.90% by August 8. After the peak, a gradual decrease was observed during the September- October period, reaching 3.13% of dry weight on October 12 (Fig. 1C).

The abundance of condensed tannins exhibited a fluctuating pattern. The initial level of condensed tannins in galls was 0.70%, which then decreased to 0.32% by August 8. After that, a gradual increase to 0.39% was observed on August 28, followed by a drop to 0.32% again on September 24. On October 12, the abundance increased to 1.92% (Fig. 1C, Table S3). Different from gallotannins, the abundance of condensed tannins was much higher in leaf samples than in gallnuts. The accumulation of condensed tannins was fast in the first two months, from 5.78% of dry leaf weight on June 21 to 12.50% on August 8. After that condensed tannin abundance gradually tapered off to 6.47% on August 28, followed by an increase to 11.34% towards the end of the season (Fig. 1C, Table S4).

### *De novo* transcriptome assembly and annotation

The Illumina sequencing data of *R. chinensis* generated 86,156,236 raw reads covering a total of 7,203,025,980 nucleotides (nt) from 15 libraries (five time points and three different types of tissues). After removing adapter sequences, ambiguous nucleotides and low-quality sequences, 80,033,622 clean reads were retained. The average length of reads was 486 nt. The clean reads were assembled into 59,522 unigenes with average length 889 and N50 1675 nt. The size statistics of assembled unigenes were shown in Supplementary Information: Tables S5, S6, and Fig. S3.

We annotated gene function of all unigenes based on common databases, including the NCBI database Non-redundant protein sequences (Nr), NCBI nucleotide sequences (Nt), the manually annotated and curated protein sequence database (Swiss-Prot), Kyoto Encyclopedia of Genes and Genomes Ortholog database (KEGG), Clusters of Orthologous Groups of proteins (COG) and Gene Ontology (GO) databases. Annotation results of unigenes are shown in Table 1.

**Table 1:** Annotation result statistics in different databases.

Database	Numbers of Unigene	Percent (%)
Nr	36,153	60.74%
Nt	31,128	52.30%
Swiss-Prot	24,856	41.76%
KEGG	21,880	36.76%
COG	14,409	24.21%
GO	28,491	47.87%

A total of 36,153 unigenes (60.74% of all unigenes) returned above-cutoff BLAST (E value < 10^−5^) results when searched against the Nr nucleotide database. The species distribution of the top hits in the Nr database showed that 23.1% of the transcriptome sequences matched with *Vitis vinifera*, followed by *Ricinus communis* (20.7%), *Populus balsamifera* (17.0%), *Amygdalus persica* (15.2%), *Fragaria vesca* (13.1%), *Glycine max* (4.2%), and *Cusumis sativus* (3.4%) (Fig. S4). In addition, 31,128 unigenes were annotated to Nt database while 24,856 unigenes were matched to proteins in Swiss-Prot (Table 1).

KEGG is a database that allows the classification of genes according to their functional categories, such as metabolic processes or cell signaling pathways. All identified sequences were subjected to KEGG to identify biological pathways relevant for gallotannin and condensed tannin synthesis. Of these, 21,880 unigenes were annotated with KEGG. In total, 437 pathways were obtained including “anthocyanin” (15 members), “isoflavonoid” (70 members), “flavone and flavonol” (118 members) and “flavonoid biosynthesis” (234 members), which are involved in gallotannin or condensed tannin synthesis and transportation (Fig. S5).

To classify orthologous gene products, 14,409 (38.19%) non-redundant unigenes were subdivided into 26 COG classifications. Among them, the cluster of “general function prediction” (4934, 34.24%) represented the largest group, followed by “transcription” (2543, 17.65%) and “replication, recombination, repair” (2307, 16.01%). The categories of “nuclear structure” (8, 0.06%) and “extracellular structures” (7, 0.05%) represented the smallest groups. The other categories are also shown in Fig. S6.

GO is a classification system to describe the properties of the genes and their products of a given organism, including three major categories as biological process, cellular component and molecular function^28^. Of the 59,522 unigenes, 28,491 non-redundant unigenes were assigned specific GO terms and categorized into 23 biological processes, 17 cellular components, and 16 molecular functions. “Metabolic process”, “cell part” and “catalytic activity” were dominant in each term (Table 1, Fig. S7).

### Genes involved in the biosynthesis of gallotannins

In order to identify *R. chinensis* transcripts that are involved in the two different tannin biosynthetic pathways, we used genome-wide transcriptome profiling to find orthologs of known tannin-producing enzymes from other plant species and create gene expression profiles that may track gallotannin peak production in galls from *R. chinensis*. For this, we generated total RNA samples from three different tissue types. These included samples from the gall itself, leaf samples from gall-bearing branches (=near a gall, YL leaf) and leaf samples from similar locations as above but from gall-free trees (NL leaf). We collected these three tissue types at eight time points during the season, identical to the dates when we conducted the gallotannin and condensed tannins measurements.

**Figure 2:**
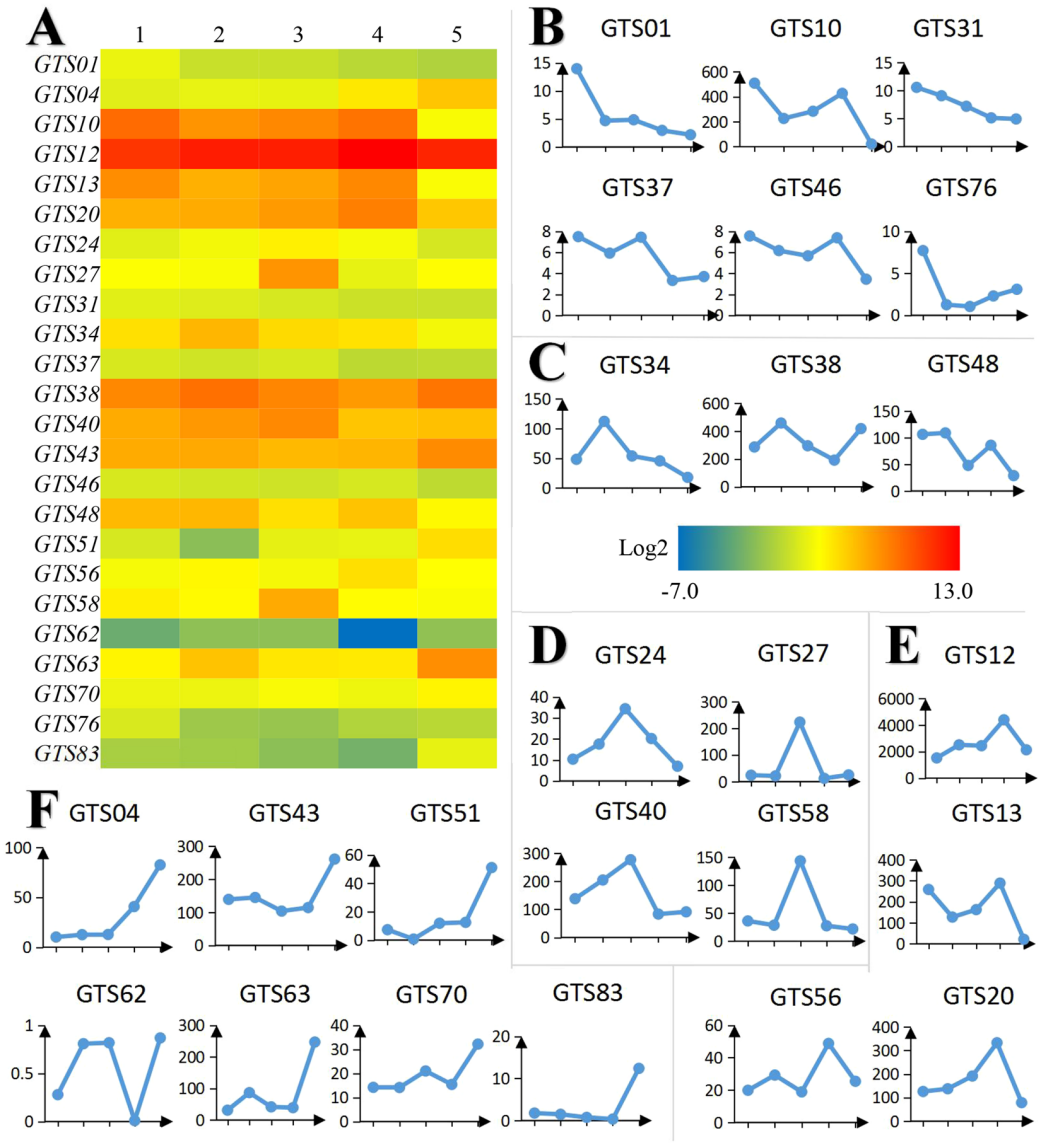
The expression profile of putative genes related to gallotannin biosynthesis. (**A**) The heatmap of expression profiles of 24 representative genes expressing profile in different developmental stage of gallnut. The horizontal 1–5 are five time point in Jun. 21, Jul. 26, Aug. 8, Aug. 18 and Oct. 12 respectively; (**B**–**F**). The different gene expression profiles of putative genes related to gallotannin biosynthesis. The different type of GTS genes peaking on June, July, August, September and October are list in (**B**,**C**,**D**,**E** and **F**), respectively. The vertical number represents RPKM for each GTS gene.

To estimate gene expression levels, read counts were normalized to Reads Per Kilo bases per Million mapped Reads (RPKM)^28^, which should reflect relative transcript abundance. RPKM > 0.3 was defined as the threshold of significant gene expression^29^. Of all 59,522 unigenes, a total of 81 unigenes related to hydrolysable gallotannins biosynthesis were identified and named as *R. chinensis gallotannins synthesis 1* (G*TS-1*) to G*TS-81* (Fig. 2A, Table S7). In the different pathways involved in the synthesis of gallotannins (Fig. S2), 6 putative unigenes encode 3-deoxy-d-arabino-heptulosonate-7-phosphate synthases [KO: K01626], 46 unigenes encodegallate 1-beta-D-glucosyltransferases [KO: K01784, K05841, K15277, K13263, K08237, K13692, K00963, K08237, K00012, K13691, ect.], 10 unigenes encode shikimate dehydrogenases [KO: K13832], 9 unigenes encode isochorismate synthases [KO: K14759], 4 unigenes encode chorismate mutase synthases [KO: K01850] and 6 unigenes encode 3-dehydroquinate synthases [KO: K01735] (Table S7).

The levels of twenty-six gene transcripts peaked on August 8, and 17 of them (65%) encode gallate 1-beta-D-glucosyltransferases. All 26 genes showed increases in gene expression from June to August, followed by a gradual decrease during September, and slight increase in October (Fig. 2D).

However, there were still 11 genes downregulated with lowest expression level on August 8, and most of them encode putative 3-deoxy-d-arabino-heptulosonate-7-phosphate synthases. In addition, the transcript levels of 14 genes peaked in June (Fig. 2B), and 5 of them (36%) encode putative gallate 1-beta-D-glucosyltransferases. The expression of 13 genes peaked in July (Fig. 2C) and 6 of them (46%) encode gallate 1-beta-D-glucosyltransferases. The expression levels of another 13 genes peaked in September (Fig. 2E), with 7 genes (54%) encoding putative gallate 1-beta-D-glucosyltransferases. The expression levels of 16 genes peaked in October (Fig. 2F), and 11 of them (69%) encodegallate 1-beta-D-glucosyltransferases.

### Genes involved in the biosynthesis of condensed tannins

**Figure 3:**
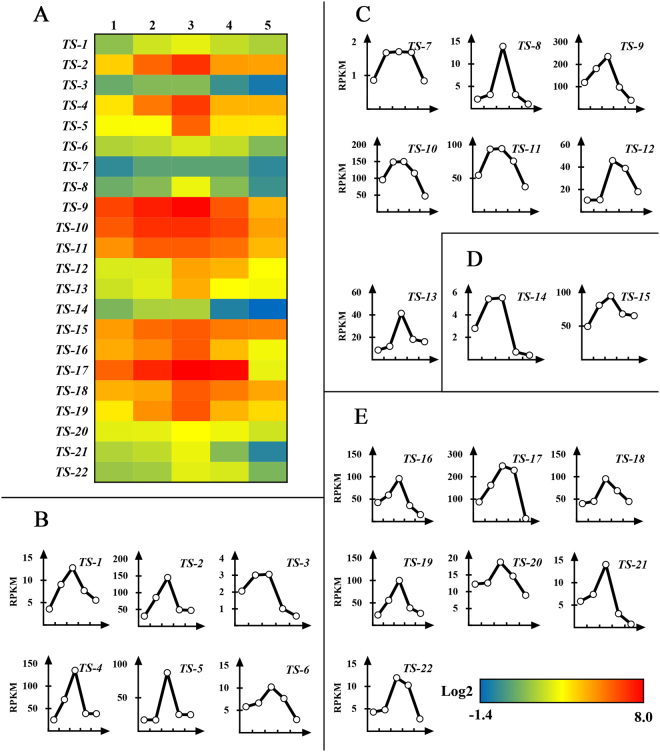
The expression profile of putative genes related to condensed tannin biosynthesis. (**A**) The hot map of 22 genes expressing profile in different developmental stage of gallnut. The horizontal 1–5 are five time points in Jun. 21, Jul. 26, Aug. 8, Aug. 18 and Oct. 12 respectively; (**B**) The change in RPKM of 6 genes involved in the pathway of flavonoid biosynthesis; (**C**) RPKM of 7 genes in flavone and flavonol biosynthesis; (**D**) RPKM of 2 genes in anthocyanin and flavanone biosynthesis; (**E**) RPKM of 7 genes in isoflavonoid biosynthesis.

Of all 437 pathways involved in the synthesis of anthocyanin, isoflavonoid, flavone, flavonol, and flavonoid (Fig. S4), 22 unigenes involved in condensed tannin biosynthesis were identified by the strategy list in Fig. S8. These genes were named as *R. chinensis condensed tannins synthesis 1* (*TS-1*) to *TS-22* (Fig. 3A) and the amino acid sequences encoded by them are listed in Dataset S1.

The 22 putative genes for condensed tannin biosynthesis have shown expression profiles consistent with changes in the abundance of condensed tannins during the five developmental phases. All 22 genes showed moderate to dramatic increases in gene expression from June to August, which was then followed by a gradual decrease during September and October (Fig. 3B–E). As shown in Table S8, we identified six unigenes linked to flavonoid biosynthesis according to the KEGG database, including *TS-1*, a putative leucoanthocyanidin reductase (LAR) [KO: K00224]; *TS-3*, a flavanone 4-reductase [KO: K13082], and four genes (*TS-2*, *TS-4*, *TS-5*, *TS-6*) that exhibited sequence similarity to leucoanthocyanidin dioxygenases (LAD). Seven putative genes (*TS-7* to *TS-13*) were involved in flavone and flavonol biosynthesis, including *TS-7* (a flavonol 3-O-methyltransferase [KO: K05279]), *TS-8* (a flavonoid 3′-monooxygenase [KO: K05280]), and *TS-9* to *TS-13* (five genes likely encoding 4-coumarate-CoA ligases (CHS) [KO: K01904, K10526]). Two genes, *TS-14* and *TS-15*, encode a putative anthocyanidin 3-O-glucosyltransferase (ACG) [KO: K13493] involved in anthocyanin biosynthesis and a phenylalanine ammonia-lyase [KO: K10775] involved in phenylpropanoid and flavanone biosynthesis (Table S8).

### Genes involved in plant defense responses

**Figure 4:**
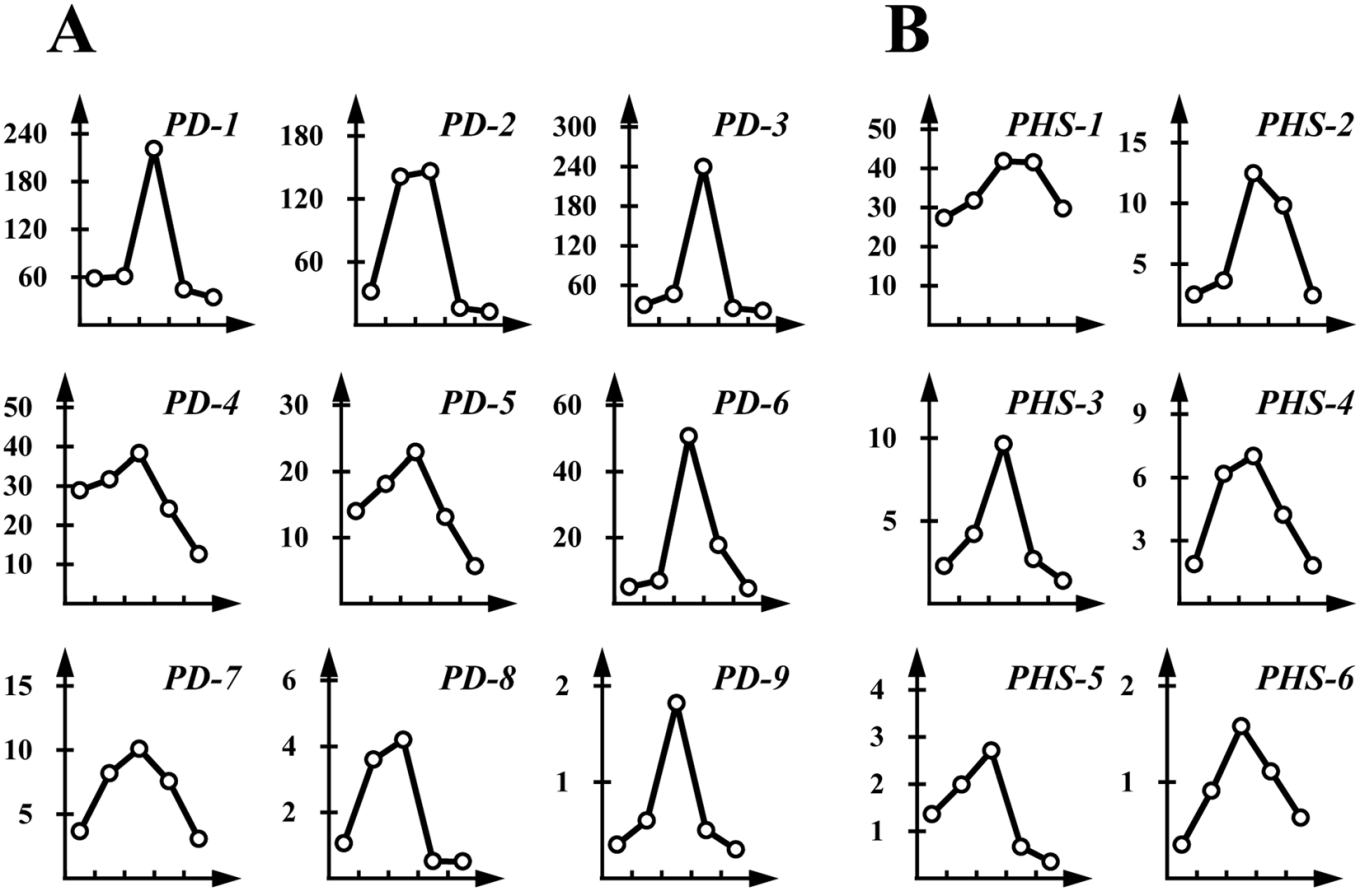
RPKM of putative genes related to plant defense and hormone biosynthesis. (**A**) The change in RPKM of 9 genes involved in plant defense at different developmental stages of gallnuts. The horizontal coordinates are five time point of Jun. 21, Jul. 26, Aug. 8, Aug. 18 and Oct. 12 respectively; (**B**) RPKM of 6 genes in the pathway of plant hormone biosynthesis.

We identified nine unigenes that are involved in plant defense. These genes were named *R. chinensisdefense system 1* (*PD-1*) to *PD-9* (Table S9, Fig. 4). Four genes (*PD-1* to *PD-4*) were related to chitinases, which have antifungal activity, and three genes (*PD-5* to *PD-7*) relate to cytochrome P450 family members, which play numerous roles including detoxification and steroid/wax synthesis. *PD-8* sequences shared similarity to a linalool synthase and *PD-9* to asqualene monooxygenase, both of which participate in the synthesis of plant terpenoids. The expression patterns of *PD-8* and *PD-9* were different from the JA-SA antagonisms found in leaf-galling phylloxera on grapes^30^. Additionally, six putative genes were identified that are involved in plant hormone biosynthesis and named as *plant hormone synthesis 1* (*PHS-1*) to *PHS-6* (Table S9), in which four genes (*PHS-2 to PHS-5*) encode adenylate isopentenyl transferases (cytokinin synthases), while the two other genes, *PHS-1* and *PHS-6*, encode a lathosterol oxidase (that regulates steroid biosynthesis) and a gibberellin 20-oxidase, respectively (Table S9). These six putative genes showed a significant increase in expression throughout gallnut development and reached the highest level on October 8. Gibberellin typically promotes intermodal growth while cytokinin induces cell division, delays senescence, and promotes bud growth in plants. These genes are likely closely related to growth processes leading to gall formation.

### Differential gene expression (DGE) analysis

**Figure 5:**
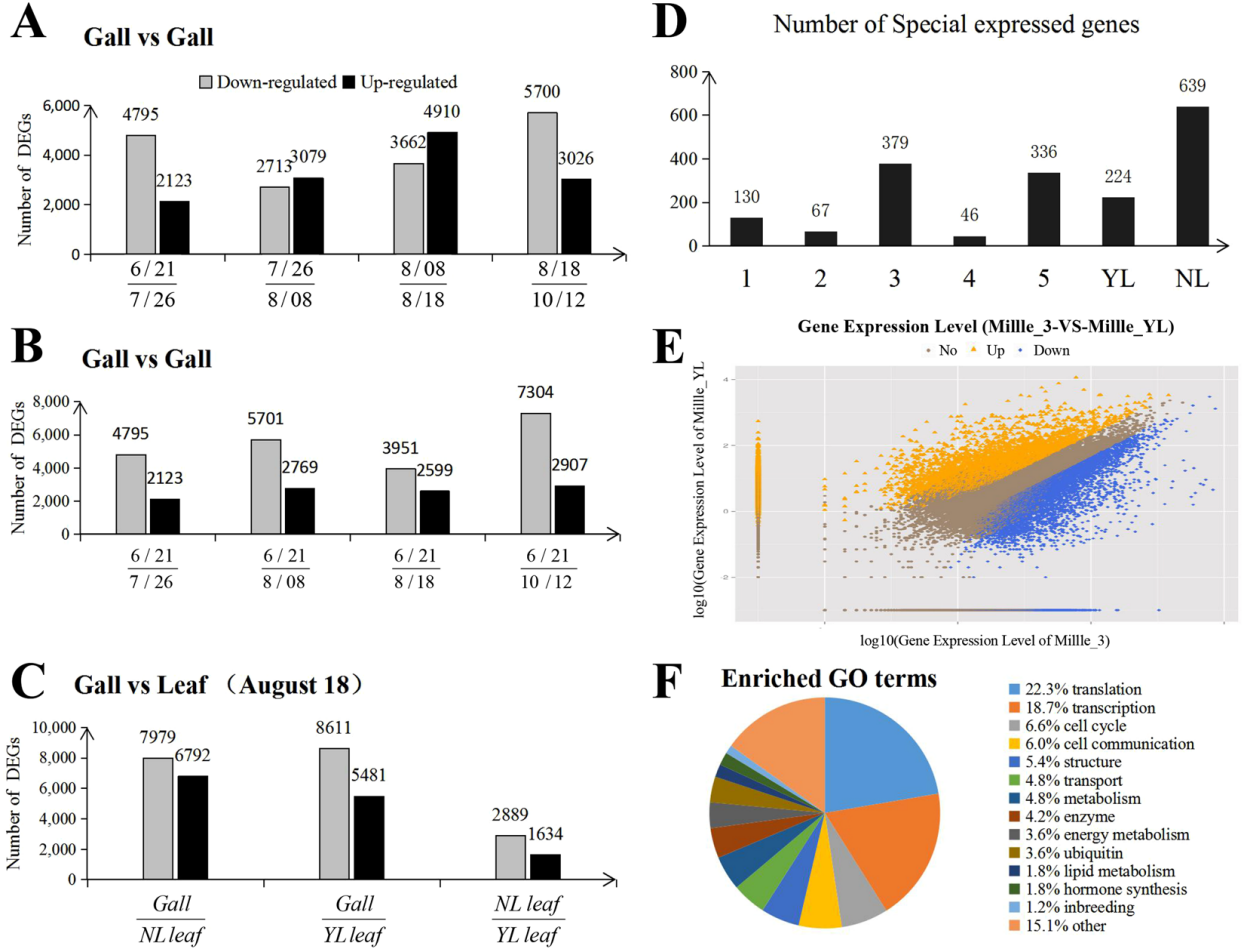
Analyses of expression profiles on different genes. (**A**,**B**) Genes expressed differentially in gallnuts at different time points. (**C**) Genes expressed differentially in gallnut YL leaf, and NL leaf samples. (**D**) The number of genes expressed specifically under each of seven different conditions. The samples with 1–5 are from gallnuts (millle_3) and leaves (millle_YL) at five different times between June 21 and October 12. (**E**) The scatter plots of all expressed genes in gallnuts compared with those in leaves. X-axis and Y-axis represent log2 values of gene expression and time points, respectively, with blue indicating down-regulation gene, orange indicating up-regulation gene, and brown indicating no change. (**F**) Gene ontology (GO) analysis of enriched functional categories. Genes were grouped into common functional categories based on GO terms.

We next did a comparative analysis of gallnut samples from five time points (6/21, 7/26, 8/08, 8/18, 10/12) with two different leaf samples (NL leaf, YL leaf) to evaluate changes across the entire transcriptome. Comparative analyses identified 5,481 genes that showed increased expression and 8,611 that showed decreased expression in the YL leaf sample on August 8 (Fig. 5A–C). Also, we identified 6,792 up-regulated and 7,879 down-regulated genes by comparing gene expression in gallnuts with the NL leaf sample at the same time points. By combinational analyses, we found 5,482 down-regulated genes among the gall and two different leaf samples. There were 1,431 genes with a higher than 10-fold expression in gallnuts compared to leaves (Fig. 5A–C), indicating that these highly enriched transcripts are gallnut-specific and take part in specific biological processes occurring in August

We used eight different conditions for specific expression analyses to identify genes expressed under certain conditions, and 379 genes were found specific to the sample collected in August (Fig. 5D, see Methods). In addition, we generated a scatter plot for KEGG enrichment results (Fig. 5E). By comparing these 379 genes with 1,431 genes that are specifically active in gallnuts, we identified 166 genes specific to the sample collected on August 8 (Fig. 5F).

To identify biological processes important for gallotannin accumulation, we performed functional gene ontology (GO)-term enrichment analysis of the gene hits identified in our screen. We found significant enrichment for multiple cellular processes, such as translation, transcription, cell cycle, and cell communication (Fig. 5F, Table S2). Intriguingly, our analysis also revealed significant enrichment of gene functions related to some special enzyme activity and synthesis (deoxyhypusine synthase, acetyltransferase, exopeptidase, tyrosine phosphatase, aminopeptidase). Furthermore, most of these highly expressed genes in *Rhus* gallnuts are involved in response to chemical stimulus and stress^31^, suggesting a specific role of these genes in interacting between aphids and host plant.

### Quantitative PCR analysis

**Figure 6:**
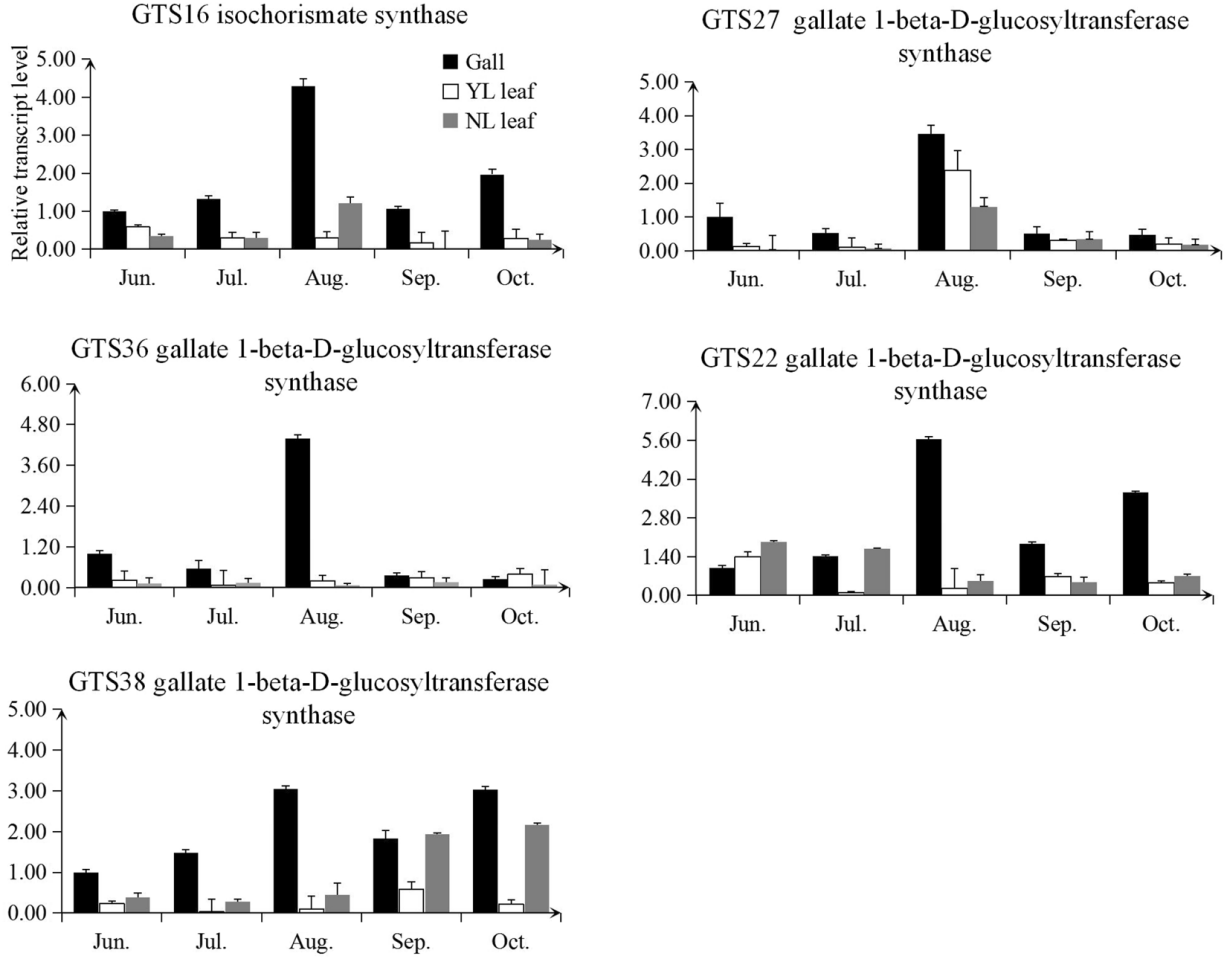
qPCR analyses of changes in transcript abundance of 5 selected genes related to gallotannin biosynthesis in *R. chinensis* at different developmental stages.

For the genes taking part in the biosynthesis of gallotannins, we chose 30 candidate genes for validation by qPCR. All of these 30 genes followed the general trend where transcript levels peaked on August 8 in gallnut samples and then started to decline. Comparing the expression profiles from leaves and gallnuts, five genes (*GTS16, GTS22, GTS27, GTS36, GTS38*) showed higher level expressionin gallnuts than in leaves. *GTS16* encode a isochorismate synthase while the other four genes encode gallate 1-beta-D-glucosyltransferases (Fig. 6). There were 20 genes with higher level expression in leaves than in gallnuts, and 17 of these genes (85%) encode gallate 1-beta-D-glucosyltransferases (Fig. S9). Additionally, five genes (*GTS04, GTS56, GTS57, GTS65, GTS66*) showed similar expression levels in both gallnut and leaf samples (Fig. S10). This suggests that gallate 1-beta-D-glucosyltransferases may play an active role in gallotannins accumulation in horned galls in response to aphid attacks.

**Figure 7:**
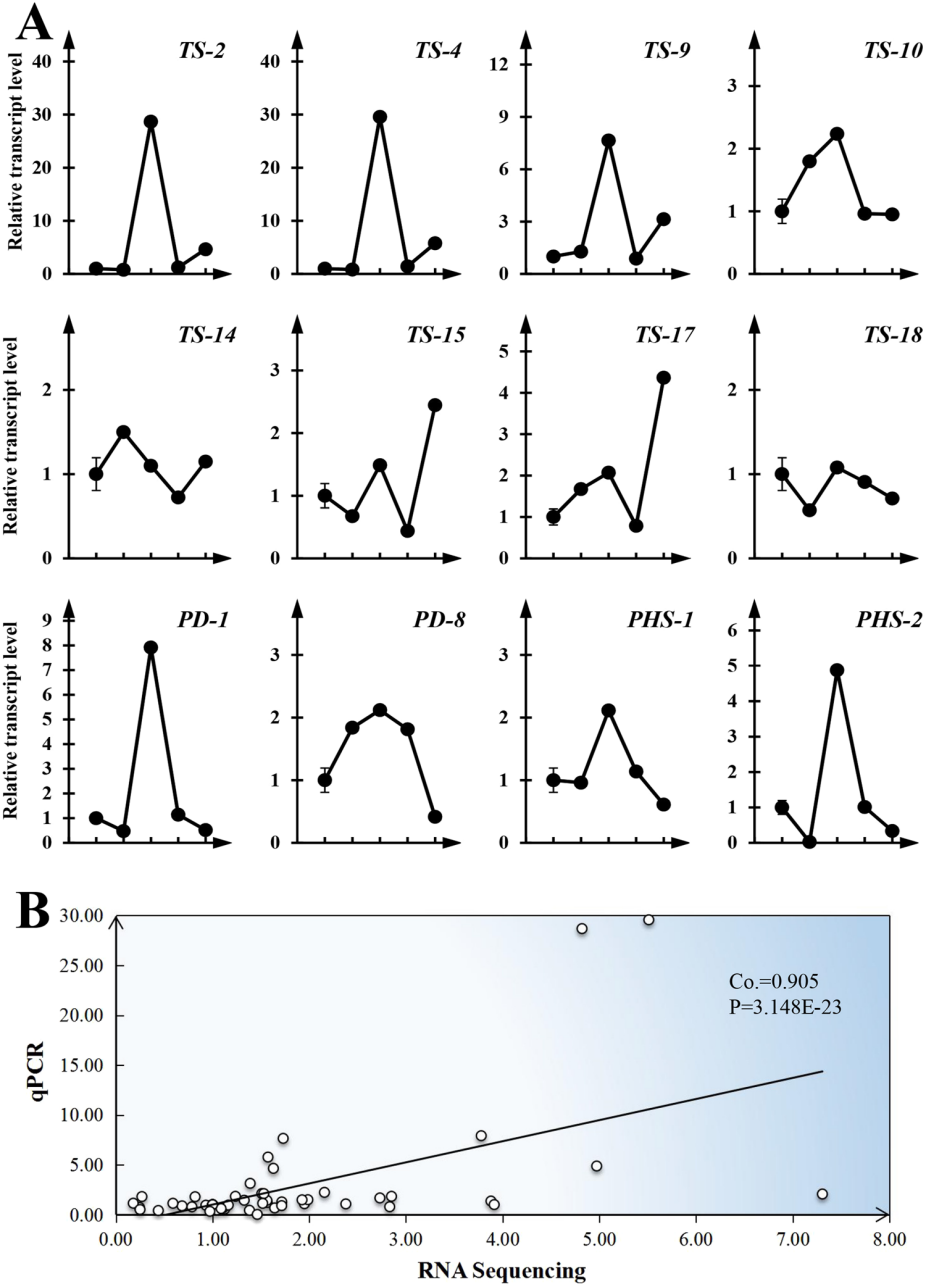
qPCR analyses of 12 selected genes in gallnuts at different developmental stages (after aphids feeding). (**A**) Line with circle is the expression profile in gallnuts. *TS-2*, *TS-4*, *TS-9*, *TS-10*, *TS-14*, *TS-15*, *TS-17* and *TS-18* are the putative genes involved in tannin biosynthesis; *PD-1* and *PD-8* represent the putative genes related to plant defense; *PHS-1* and *PHS-2* represent the genes related to plant hormone synthesis. Horizontal axis indicates sample collection time (Jun. 21, Jul. 26, Aug. 8, Aug. 18 and Oct. 12, respectively). (**B**) The correlation between qRT-PCR and RPKM data.

We selected eight genes with putative roles in condensed tannin biosynthesis for validation via qPCR (Fig. 7A). In gallnuts, the condensed tannin biosynthesis (*TS*) genes, followed the general trend we observed in the RNA-Seq data with transcript levels peaking on August 8, followed by a decrease until October 12, the last time point. However, *TS-14* gene showed the highest transcript abundance on July 26 followed by decrease till October 12. And the relative expression of the *TS-15* did not follow this trend but displayed the highest expression on October 12. For the plant defense genes *PD-1* and *PD-8*, we found the similar results to the RNA-Seq, with peak expression on August 8, followed by a decline until the end of the season. For the plant hormone biosynthesis genes *PHS-1* and *PHS-2*, transcript levels again peaked on August 8, and then declined afterwards (Fig. 7A). A comparison of the qPCR results with the corresponding RNA sequencing data resulted in a correlation coefficient of 0.905 with P = 3.148 × 10^−23^ (Fig. 7B).

## Discussion

Due to their high gallotannin content, Chinese gallnuts have been used for medical and chemical purposes for more than two millennia^8,32^. Although the biological characteristics of galling aphids have been widely studied, the mechanisms of tannin accumulation in gallnuts by insect stimulus have remained unclear. Next generation sequencing (NGS) technology offers convenient tools for research on functional genomics, comparative genomics and genetic analysis of non-model organisms^31^. Hitherto, no genomic data on *R. chinensis* has been reported. RNA-Seq provides a unique opportunity to identify genes acting in pathways at specific times of development. Here we examined several time points during a single growth season to track transcript profiles in maturing horned galls from *R. chinensis*. This approach also allows the *de novo* assembly of a *R. chinensis* transcriptome, which we then used to identify genes associated with hydrolysable and condensed tannins biosynthetic pathways.

In this study, we analyzed the transcriptome of *R. chinensis* and identified 59,522 unigenes with 60.74% of them returned above-cutoff BLAST results. When we compared the *R. chinensis* transcriptome to those from other plant species, we found that it was most similar to *Vitis vinifera* (Fig. S4). The possible reason for this similarity may be due to the fact that both species produce high levels of gallotannins and thus may share many pathways related to tannin biosynthesis. Upon examination, 28,491 unigenes were annotated to GO, 14,409 to COG, and 21,880 to KEEG. The annotated transcript sequences provide an invaluable resource for the identification of genes linked to tannin biosynthesis.

A total of 81 unigenes related to hydrolysable tannin biosynthesis were identified (*GTS-1* to *GTS-81*), and 46 unigenes (57%) encode gallate 1-beta-D-glucosyltransferases. Twenty-six gene transcript levels peaked on August 8, among which 17 (65%) encode gallate 1-beta-D-glucosyltransferases (Table S7). This observation suggests that the gallate 1-beta-D-glucosyltransferases may play a vital role in the pathway of gallotannin synthesis. The gallotannin content of horned gallnuts could be enhanced for practical application in the future once we can manipulate or over-express the genes that encode gallate 1-beta-D-glucosyltransferases and other target genes in crucial steps of gallotannin biosynthesis. The use of engineering techniques would be a cost effective way for the production of natural active substances, including tannins, in plants.

In our research, all the 30 genes potentially involved in hydrolysable tannin biosynthesis exhibited highest expression on August 8 based on both qPCR and transcriptomic data. This is consistent with accumulation of gallotannins in gallnuts. Twenty-two putative genes involved in the synthesis of anthocyanins, isoflavonoids, flavones, flavonols and flavonoids also displayed expression profiles consistent with changes in condensed tannins content during gallnut maturation (Figs 2 and 3). The expression profiles for all the 81 genes putatively involved inhydrolysable tannin biosynthesis also reached the highest levels on August 8. Our data indicate that August in Yunnan is the best time for gall collection to extract tannins.

Nine genes encoding potentially components in plant defense pathways have also exhibited the highest expression levels on August 8, including genes related to chitinases involved in plant antifungal activity, cytochrome P450s with roles in a plethora of processes such as wax and cuticle synthesis as well as detoxification pathways, a linalool synthase and squalene monooxygenase that have roles in the synthesis of plant terpenoids (Table S9). The expression of these genes increased strongly from June to August, followed by a gradual decrease during September and October (Fig. 3). The similar expression pattern of plant defense genes and genes involved in tannin biosynthesis is not surprising since the production of tannins by plants might also be a defense reaction against insects^33,34^. Nevertheless, the impact of tannin on aphid gall fundatrices remains to be determined case by case. There are reports that suggest the abundance of tannins in gall tissues correlates positively with reproductive success of galling aphid fundatrices^35^. This might be due to the fact that during the long term coevolution, gall aphid has gained the ability to utilize tannins for its benefit.

Our study focused on the different tannin biosynthesis in gallnuts of *R. chinensis* and provides a foundation for understanding the regulation of how tannin production is regulated and how it responds to the complex interactions between host plants and herbivores. Knowledge of the complex plant-herbivore interactions on a molecular level is helpful to improve the understanding of interaction with plants against herbivores and the co-evolution strategies between aphids and their host plants.

## Materials and Methods

### Sample collection

The aphid *S. chinensis* was reared on sumac trees (*R. chinensis*) in the Research Institute of Resources Insects of Kunming, Yunnan province, southwest China. The horned galls induced by *S. chinensis* from each developmental stage were collected once every fifteen days, resulting in eight samples. After removing aphids from the gall tissues, we divided the collected galls into two parts. One part was frozen in liquid nitrogen and stored at −80 °C for later isolation of RNA. We used the other part for the determination of tannins concentrations. Leaf samples from gall-bearing branches (=near a gall, YL leaf) and leaf samples from similar locations as above but from a gall-free trees (NL leaf) was collected in 8/08. The sample was frozen in liquid nitrogen immediately until use.

### Tannin measurements

Gallotannin and condensed tannin concentrations in gallnut and leaf samples from *R. chinensis* were measured as described previously^36–38^. Catechin was used as the standard for estimating the weight percentage of condensed tannins and absorbance was measured at 500 nm. For hydrolysable tannins, tannic acid was used as the standard and absorbance was measured at 276 nm.

### Illumina sequencing and transcriptome analysis

After washed three times in DEPC-treated (diethylpyrocarbonate) water, 100 mg gallnut tissues of each developmental stage were used to isolate total RNA separately using an RNA Extraction Kit (BioTeke Corporation, Beijing, China) according to the manufacturer’s protocols. RNA purity and integrity were assessed using a RNA Nano 6000 Assay Kit (Agilent Technologies, CA, USA). First strand cDNA was synthesized with random hexamers as primers and mRNA fragments as templates. The double-stranded cDNA was synthesized and purified using a QiaQuick PCR Extraction kit (QIAGEN, MD, USA) and washed using EB buffer for end repair and the addition of single adenines. Sequencing adaptors were ligated to the fragments, and the required fragments were purified by agarose gel electrophoresis and enriched by PCR amplification. Sequencing libraries were generated using a Next Ultra Directional RNA Library Prep Kit from Illumina (New England Biolabs, MA, USA). Products were purified with the AMPure XP system (Beckman Coulter, CA, USA) and library quality was confirmed on the Agilent 2100 Bioanalyzer (Agilent Technologies, CA, USA). A total of 15 transcriptomic libraries (five time points and three different types of tissues) were made in this study. After cluster generation, libraries were sequenced on Illumina HiSeq2000 platform (BGI, Shenzhen, China) and paired-end reads were generated.

To obtain clean reads, reads with adapters with more than 10% unknown bases and other low-quality reads were removed before data analysis. Transcriptome assembly was accomplished using Trinity with default parameters^39^. We assessed sequences by read quality, statistics of alignment analysis, sequencing saturation analysis, distribution of reads on the reference gene, and distribution of reads on the reference genome.

### Gene annotation

Gene function of all assembled unigenes was annotated in the NCBI database Non-redundant protein sequences (Nr), NCBI nucleotide sequences (Nt), the manually annotated and curated protein sequence database (Swiss-Prot), Kyoto Encyclopedia of Genes and Genomes Ortholog database (KEGG), Clusters of Orthologous Groups of proteins (COG) and Gene Ontology (GO) databases.

### Condition specification for gene expression analysis

Conditions for specific expression analysis were used to identify gene expression in certain conditions.

1 $${P}_{i}(g)=\sum _{x={e}_{i}(g)}^{E(g)}(\begin{array}{c}E(g)\\ x\end{array}){p}_{i}^{x}{(1-{p}_{i})}^{E(g)-x}$$

Ei(g) is the number of reads of gene g in sample i, so the total number of reads of gene g in all samples is E(g) = ∑iei(g), assuming that si is the number of all reads in sample i. The expectations of each gene’s reads in the sample i is proportional to the pi = si/∑isi. For gene g, if evenly expressed in all samples, its read number in sample i is fi = E(g)pi. We defined enrichment expression as (EE)EEi = ei(g)/fi(g), and it represented the proportion of expected observations of gene g in sample i. A greater EE value represented the gene that was more favorably expressed in sample i. In order to assess if a larger EE value is due to accidental factors rather than a real expression, we defined a P value for the enrichment of expression, and it is given in the formula.

### Quantitative real-time PCR (qPCR) analysis

To validate the result of the RNAseq analysis, twelve genes were selected from *Rhus* trees transcriptome data to perform qPCR. Amplification was performed with a SG Fast qPCR Master Mix (Sangon Biotech, Shanghai, China) on the BIO-RAD CFX96 TM Real-Time system (BIO-RAD, CA, USA) and the relative standard curve method was adopted to analyze the relative expression levels of the genes. Each reaction was carried out with 2 µl of a 1/40 (v/v) dilution of the first cDNA strand, 0.5 mM of each primer in a total volume of 20 µl. The cycling conditions were: 95 °C for 3 min followed by 40 cycles of denaturation at 95 °C for 7 s, annealing and extension at 57–60 °C, depending on the primer set (S10), for 45 s. At the end of the reaction, PCR amplification specificity was verified by obtaining a dissociation curve, derived by cooling the denatured samples to 55 °C and raising the temperature 0.5 °C for 10 s for each cycle, for a total of 80 cycles until reaching 95 °C. The PCR products were also analyzed on 1.5% agarose gels, and subsequently purified and sequenced to confirm faithful amplification. Actin was selected as a reference for normalization of template concentration. Three independent biological replicates were used for each condition.

### Data availability

Raw sequence reads have been submitted to NCBI Sequence Read Archive (BioprojectPRJNA338376). The assembled unigenes of the gall, leaf samples from gall-bearing branches (YL leaf) and leaf samples gall-free trees (NL leaf)can be found in SRA: (https://www.ncbi.nlm.nih.gov/sra). Sequences of the nonredundant assembly transcripts, the annotation (fasta file)can be found in SRA: (https://www.ncbi.nlm.nih.gov/sra/?term=PRJNA338376).

## Electronic supplementary material

Supplementary Information
