# Supplementary material for: Molecular mechanisms of tannin accumulation in Rhus galls and genes involved in plant-insect interactions
Source: Sci Rep. 2018 Jun 29;8:9841. doi: 10.1038/s41598-018-28153-y (PMC6026138; doi:10.1038/s41598-018-28153-y)
Supplement: Supplementary file 1 — Supplementary Information [file 41598_2018_28153_MOESM1_ESM.pdf]

Supplementary Information

Molecular mechanisms of tannin accumulation in Rhus galls and genes involved in plant-insect interactions

Hang Chen[1,2*], Juan Liu[1], Kai Cui[1], Qin Lu[1], Chao Wang[1,3], Haixia Wu[1], Zixiang Yang[1], Weifeng Ding[1], Shuxia Shao[1], Haiying Wang[1], Xiaofei Ling[1], Kirst King-Jones[4], Xiaoming Chen[1,2*]

[1] Research Institute of Resource Insects, Chinese Academy of Forestry, Kunming, China.

[2] The Key Laboratory of Cultivating and Utilization of Resources Insects, State Forestry Administration, Kunming, China.

[3] Southwest Forestry University, Kunming, China.

[4] Department of Biological Sciences, Biological Sciences Building, University of Alberta, Edmonton, Canada.

[*]Corresponding author: Xiaoming Chen and Hang Chen, Research Institute of Resource Insects, Chinese Academy of Forestry, Kunming, China.

Telephone: +86 0871 63860019

E-mail: Cafcxm@139.com, stuchen6481@gmail.com

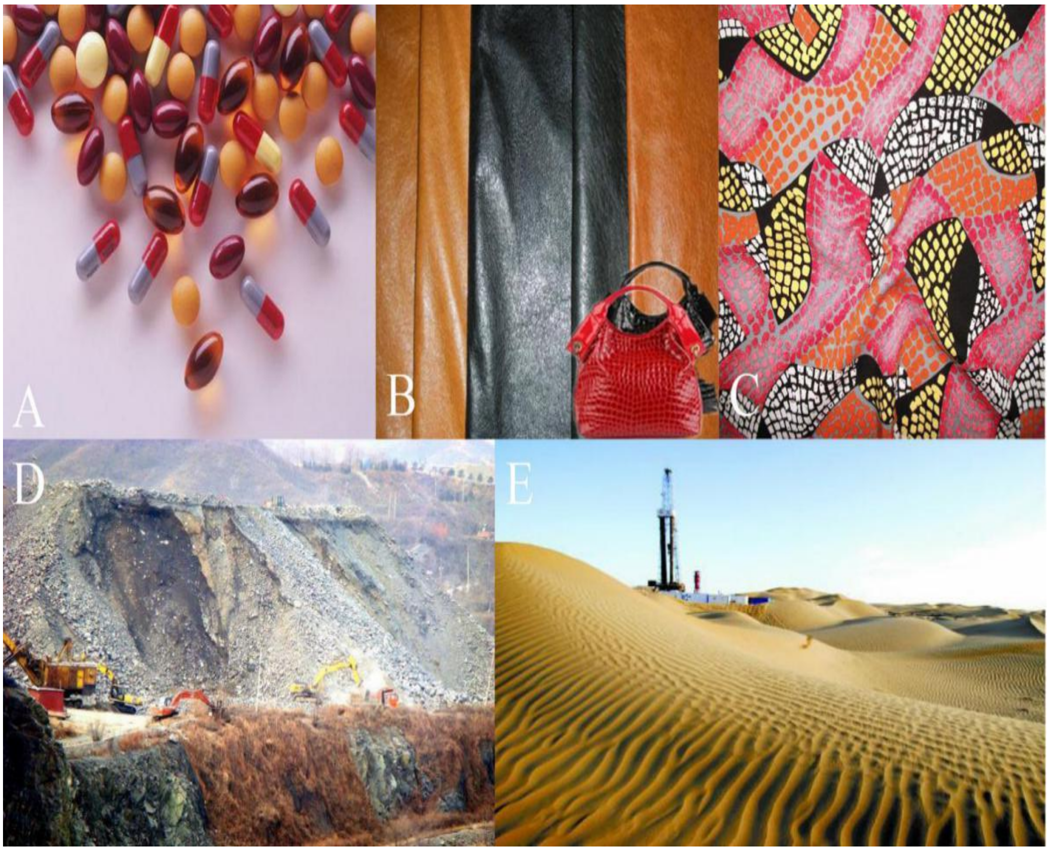

Figure S1. Utilization of tannic acid derived from gallnut of *R. chinensis*. A. Medicine (synergist); B. Leatherworking; C. Textile printing; D. Mineral separation; E. Oil exploration.

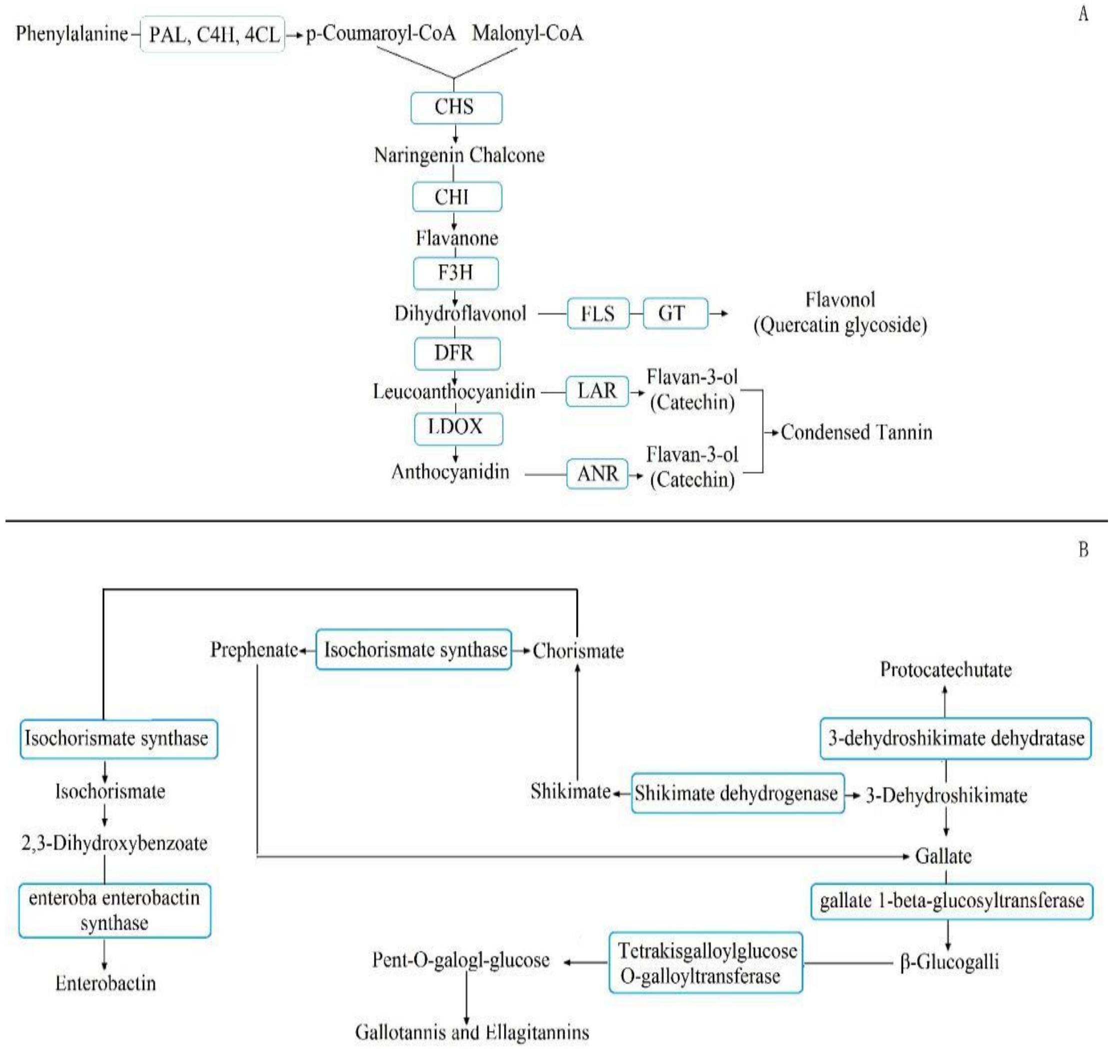

Figure S2 The pathways of Tannins biosynthesis. A. The procession of condensed tannin biosynthesis, PAL (phenylalanine ammonialyase); C4H (cinnamate 4-hydroxylase); 4CL (4-coumarate CoA ligase); CHS (chalcone synthase); DFR (dihydroflavonol 4-reductase); LAR (leucoanthocyanidin reductase); ANR (anthocyanidin reductase); **B. The procession of gallotannin biosynthesis.**

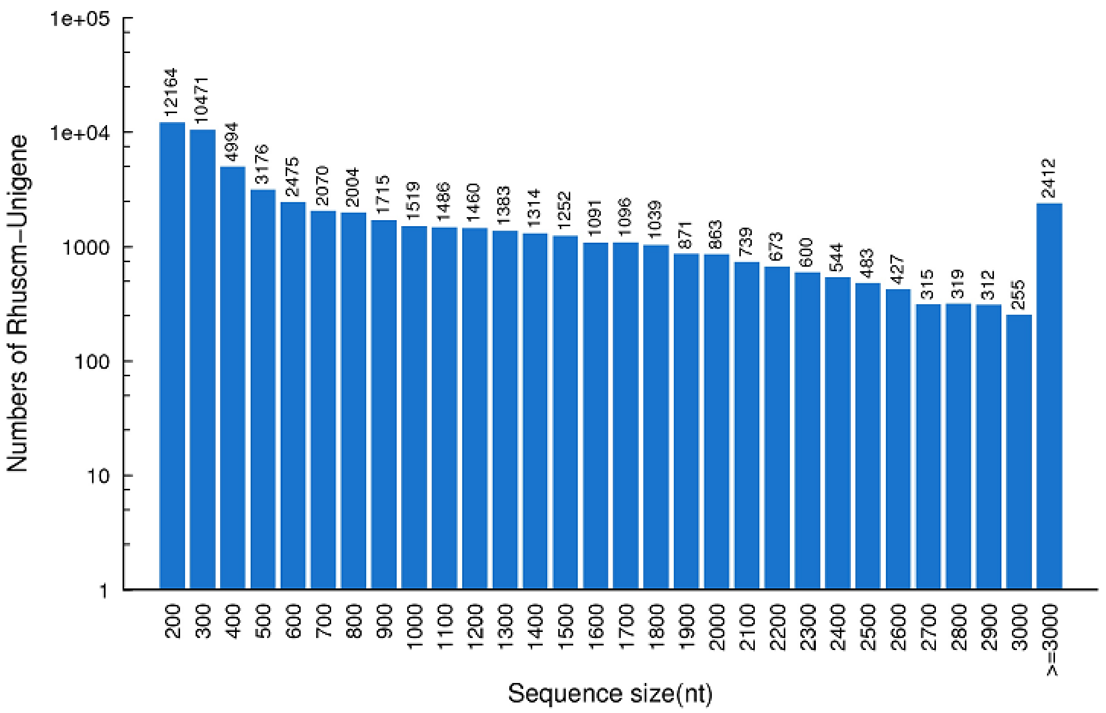

Figure S3 Length distribution of unigenes of *R. chinensis*

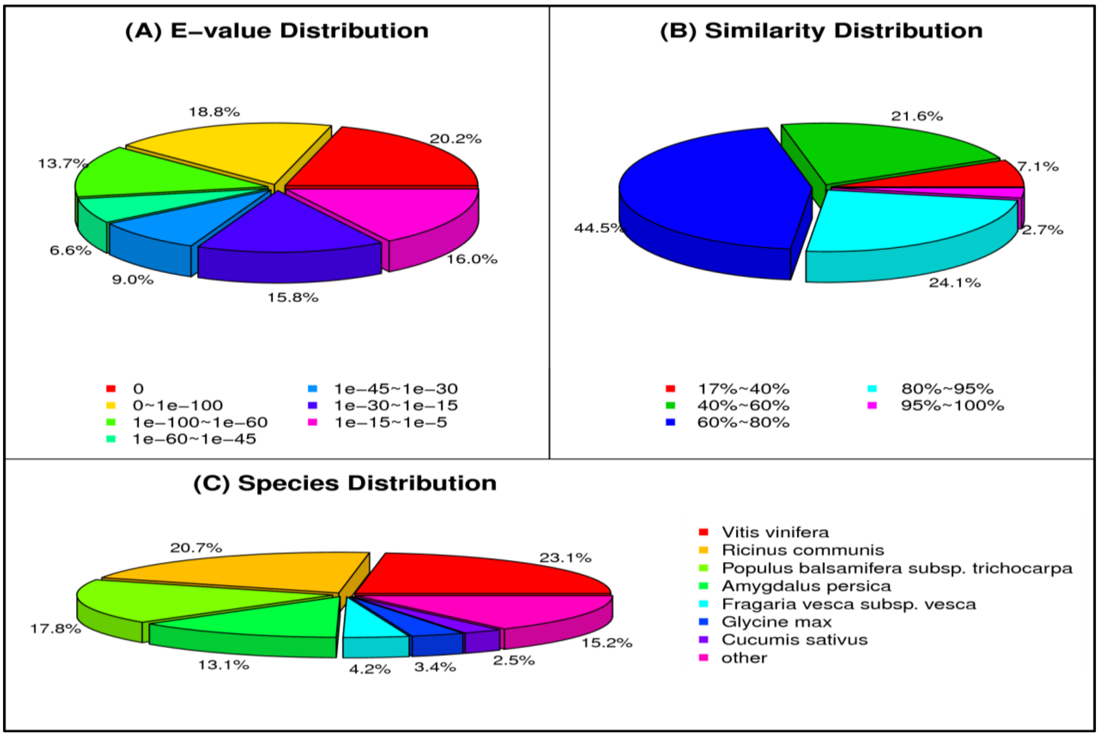

Figure S4. Data of Nr classification. (A) The E-value distribution of the result of Nr annotation. (B) The similarity distribution of the result of Nr annotation. (C) The species distribution of the result of Nr annotation.

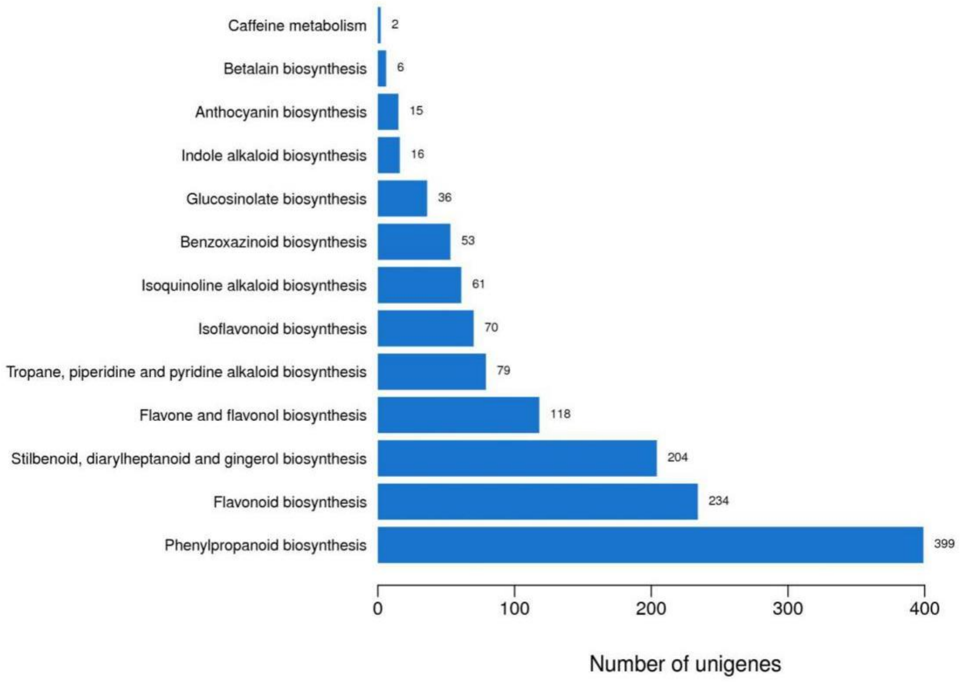

Figure S5. Pathway on biosynthesis of other secondary metabolites by KEGG. X-axis is the number of unigenes which annotated each pathway and y-axis is the sub-pathway.

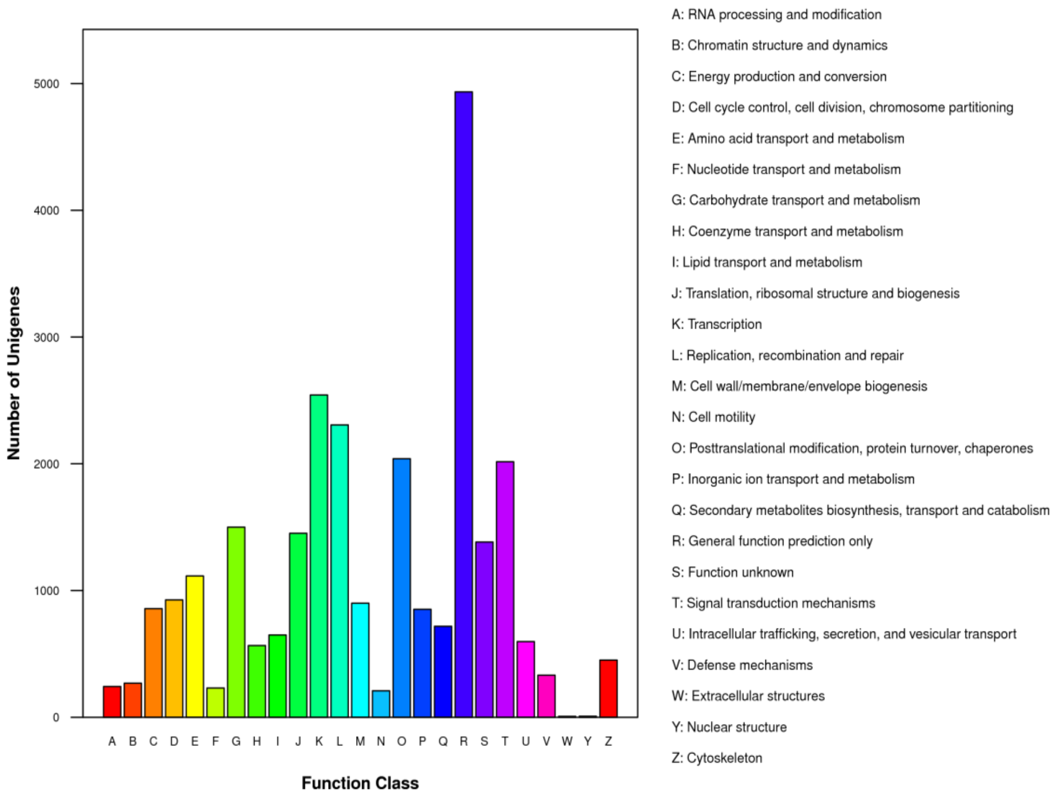

Figure S6. COG function classification of Unigenes. The horizontal coordinates are function classes of COG and the vertical coordinates are numbers of Unigenes in one class. The notation on the right is the full name of the functions in X-axis.

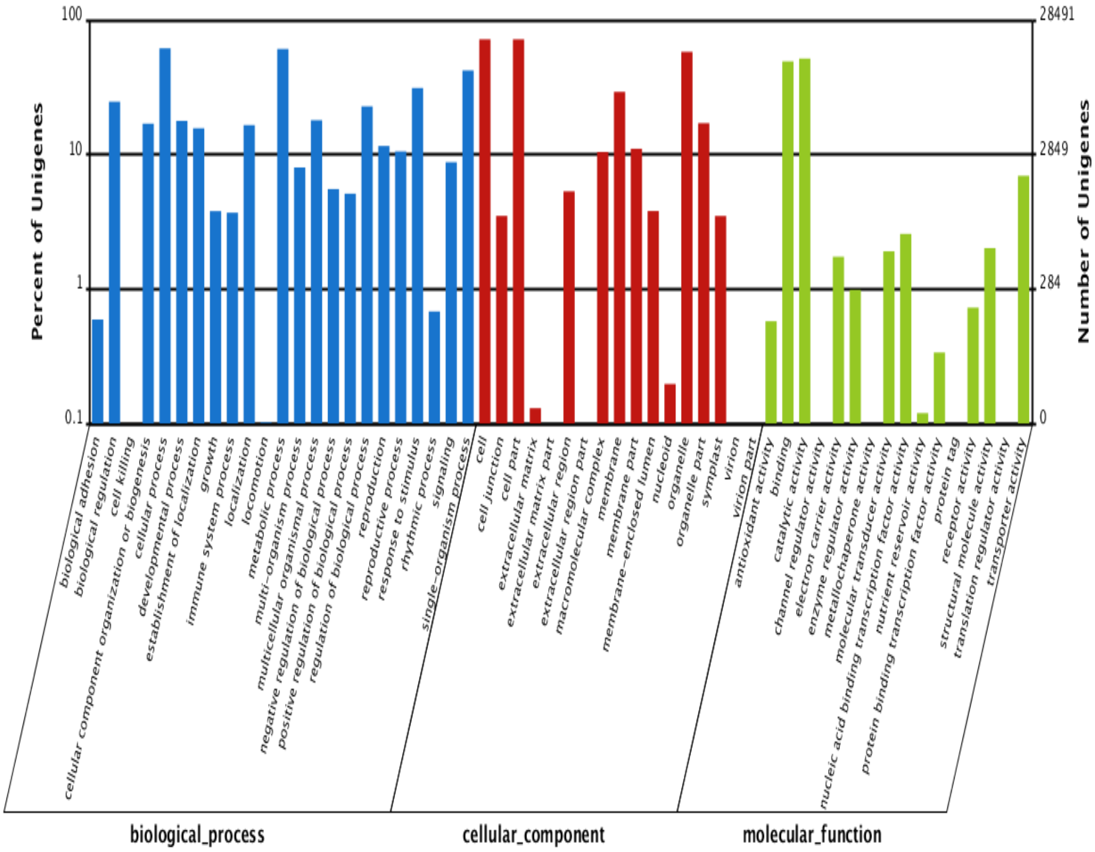

Figure S7. GO classification analysis of Unigenes in All-Unigene. GO functions is showed in X-axis. The right Y-axis shows the number of genes which have the GO function, and the left Y-axis shows the percentage.

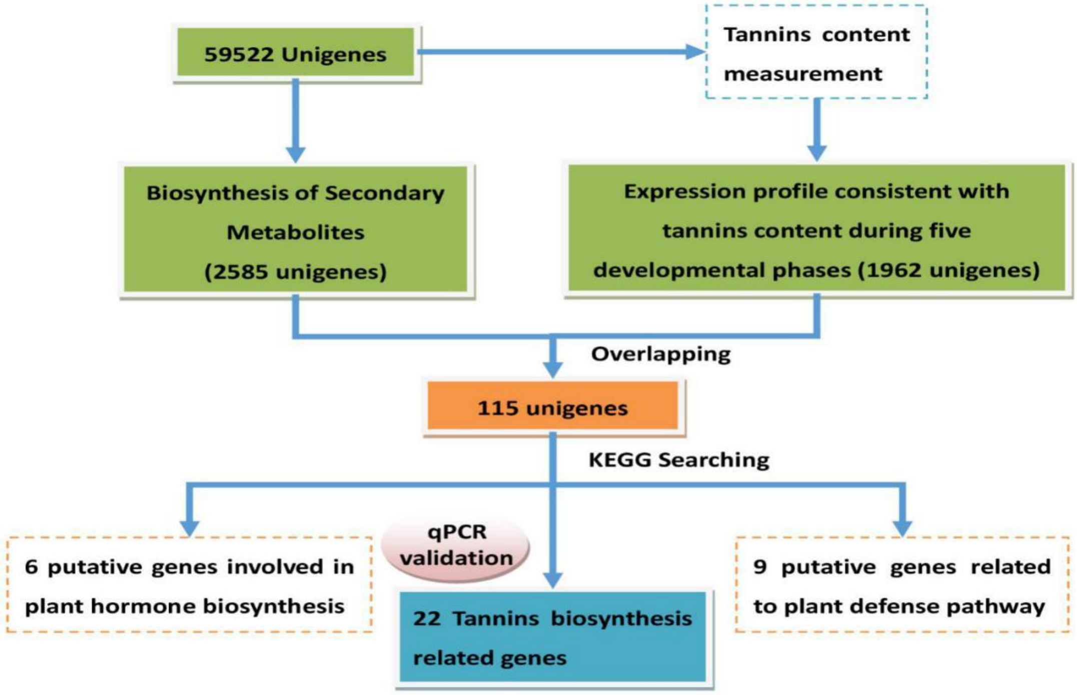

Figure S8. Selective strategy of putative genes related to condensed tannin biosynthesis, plant defense and hormone pathway from the whole unigenes of transcriptome data.

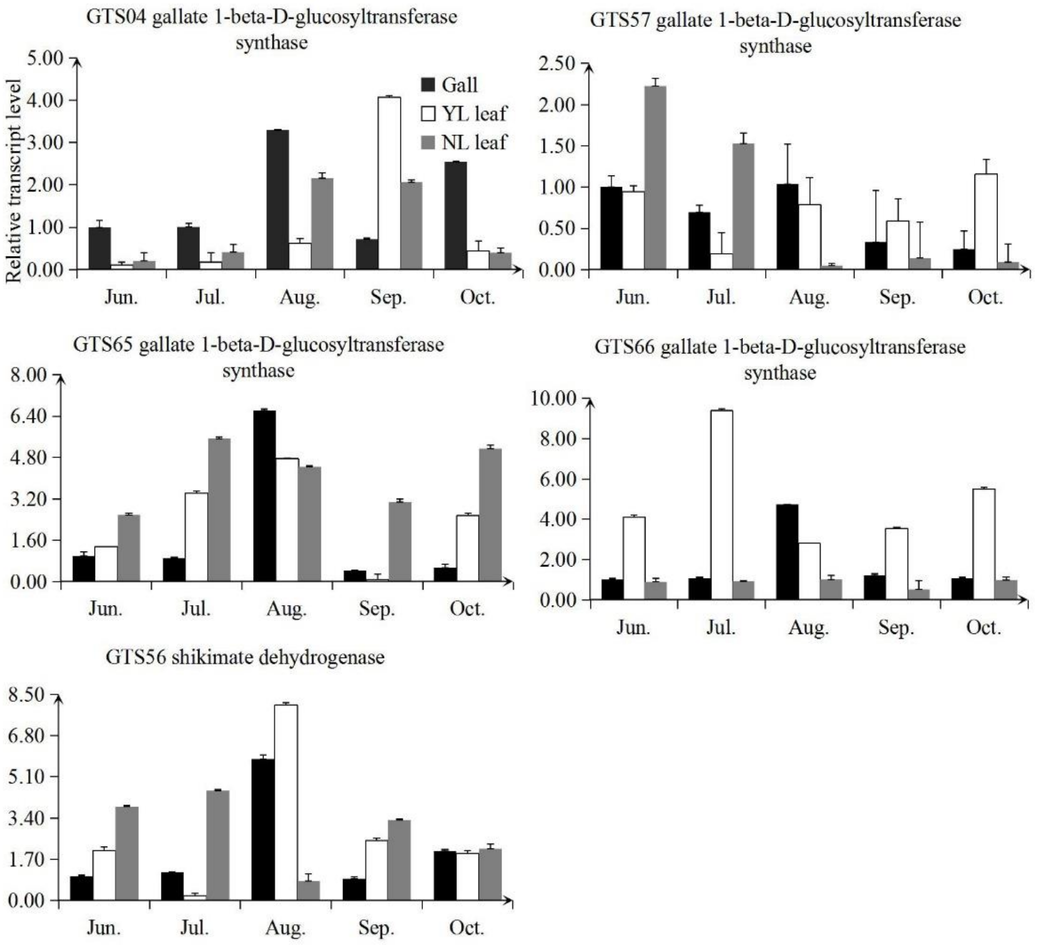

Figure S9. qPCR analyses of changes in transcript abundance of 5 putative genes related to gallotannin biosynthesis of *R. chinensis* in different developmental stage showing similar levels in gallnut than in leaf.

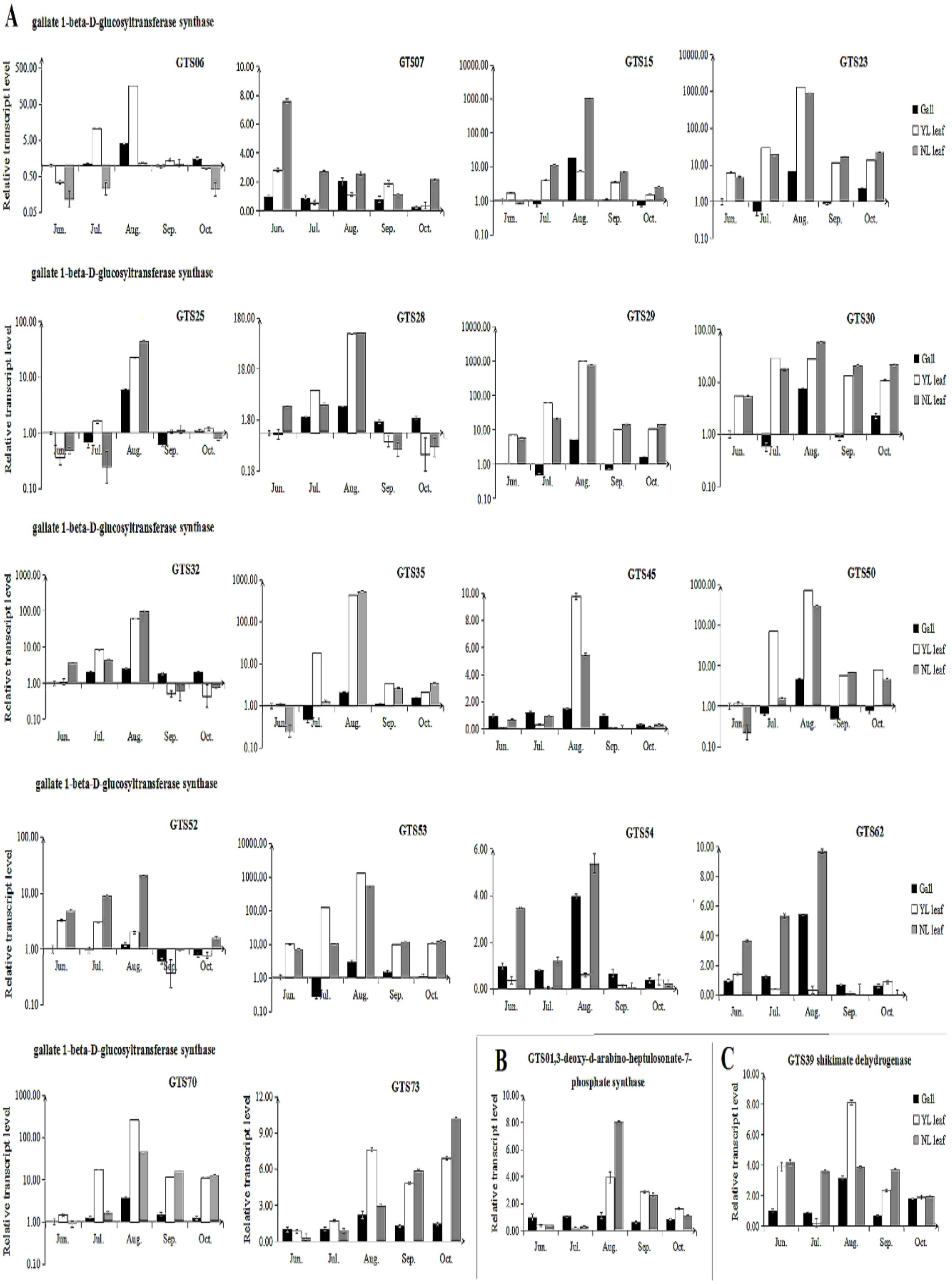

Figure S10. qPCR analyses of changes in transcript abundance of 20 putative genes related to gallotannin biosynthesis of *R. chinensis* in different developmental stage show higher levels in leaf than in gallnut.

Table S1 The weight percentage of tannin in gallnut during different developmental stage of *Rhus chinensis*

	Jun.21	Jul.26	Aug.8	Aug.18	Aug.28	Sep.10	Sep. 24	Oct.12
**1**	0.3882	0.7075	0.7279	0.7005	0.6823	0.6825	0.6559	0.5937
**2**	0.3736	0.5819	0.7621	0.7179	0.6538	0.6536	0.5874	0.5840
**3**	0.3883	0.6780	0.7537	0.7224	0.7083	0.6769	0.6419	0.5871
**Ave.**	0.3834	0.6558	0.7479	0.7136	0.6815	0.6710	0.6284	0.5883
**SE**	0.0085	0.0657	0.0178	0.0116	0.0273	0.0153	0.0362	0.0049

Table S2 The weight percentage of tannin in leaf during different developmental stage of *R. chinensis*

	Jun.21	Jul.26	Aug.8	Aug.18	Aug.28	Sep.10	Sep. 24	Oct.12
**1**	0.0272	0.0317	0.0389	0.0170	0.0280	0.0335	0.0361	0.0303
**2**	0.0293	0.0301	0.0435	0.0233	0.0314	0.0276	0.0435	0.0332
**3**	0.0275	0.0457	0.0646	0.0200	0.0338	0.0338	0.0423	0.0303
**Ave.**	0.0280	0.0359	0.0490	0.0201	0.0311	0.0316	0.0406	0.0313
**SE**	0.0019	0.0086	0.0137	0.0032	0.0029	0.0035	0.0040	0.0017

Table S3 The weight percentage of condensed tannin in gallnut during different developmental stage of *Rhus chinensis*

	Jun.21	Jul.26	Aug.8	Aug.18	Aug.28	Sep.10	Sep. 24	Oct.12
**1**	0.68%	0.36%	0.31%	0.38%	1.19%	0.70%	0.55%	1.77%
**2**	0.64%	0.35%	0.30%	0.35%	0.95%	0.63%	0.55%	1.79%
**3**	0.78%	0.34%	0.32%	0.37%	1.05%	0.65%	0.47%	1.86%
**Ave.**	0.70%	0.37%	0.32%	0.42%	0.90%	0.67%	0.39%	1.92%
**SE**	0.07%	0.01%	0.01%	0.01%	0.12%	0.03%	0.05%	0.05%

Table S4 The weight percentage condensed tannin in leaf during different developmental stage of *R. chinensis*

	Jun.21	Jul.26	Aug.8	Aug.18	Aug.28	Sep.10	Sep. 24	Oct.12
**1**	6.63%	10.21%	12.86%	8.65%	6.57%	8.05%	10.38%	11.64%
**2**	5.36%	9.67%	12.29%	9.12%	6.78%	8.29%	9.99%	11.68%
**3**	5.34%	9.24%	12.34%	7.79%	6.06%	7.42%	11.08%	10.69%
**Ave.**	5.78%	9.71%	12.50%	8.52%	6.47%	7.92%	10.48%	11.34%
**SE**	0.74%	0.48%	0.31%	0.68%	0.37%	0.45%	0.55%	0.56%

Table S5 Output statistics of sequencing

Samples	Total Raw Reads	Total Clean Reads	Total Clean Nucleotides (nt)	Q20 percentage	N percentage	GC percentage
**Rhuscm**	86,165,236	80,033,622	7,203,025,980	97.85%	0.00%	43.73%

Table S6 Statistics of assembly quality

	Sample	Total Number	Total Length(nt)	Mean Length(nt)	N50	Total Consensus Sequences	Distinct Clusters	Distinct Singletons
**Contig**	Rhuscm	88,592	43,027,436	486	1224	-	-	-
**Unigene**	Rhuscm	59,522	52,899,372	889	1675	59,522	15,125	44,397

Table S7. RPKM of 81 putative genes related to gallotannin biosynthesis

Gene name	Jun.	Jul.	Aug.	Sept.	Oct.	Average	Functional categories
**GTS38**	286.62	459.01	295.53	192.21	418.97	330.47	
**GTS17**	110.79	202.42	244.97	81.79	136.13	155.22	
**GTS43**	139.36	145.16	103.95	114.59	261.29	152.87	
**GTS22**	75.28	158.56	155.61	80.14	119.59	117.84	
**GTS63**	31.36	86.94	41.88	39.41	247.34	89.39	
**GTS15**	29.66	107.54	270.72	6.71	21.84	87.29	
**GTS30**	62.91	71.56	209.98	13.70	32.90	78.21	
**GTS27**	24.10	21.42	223.27	12.25	25.20	61.25	
**GTS23**	50.83	43.13	129.54	6.25	35.20	52.99	
**GTS58**	36.07	28.49	143.29	27.43	21.77	51.41	
**GTS67**	58.90	66.30	35.16	32.40	57.46	50.04	
**GTS53**	30.16	22.21	82.57	5.44	51.33	38.34	
**GTS04**	10.19	12.56	12.76	40.67	82.41	31.72	
**GTS35**	35.21	6.14	53.60	10.82	36.99	28.55	
**GTS41**	32.56	6.48	51.70	13.89	37.60	28.45	
**GTS11**	11.07	14.04	16.05	38.60	52.48	26.45	
**GTS66**	19.31	16.89	30.71	20.79	40.96	25.73	UDP-glucose
**GTS14**	8.83	4.06	88.54	2.34	12.26	23.21	
**GTS42**	23.34	25.31	17.45	18.91	17.49	20.50	
**GTS70**	14.24	14.17	21.05	15.46	32.23	19.43	
**GTS18**	13.58	20.24	22.43	29.90	9.37	19.10	
**GTS45**	15.78	14.61	16.41	25.75	16.24	17.76	
**GTS52**	18.53	14.38	24.96	11.05	3.66	14.52	
**GTS50**	11.90	1.78	25.47	5.37	16.00	12.10	
**GTS32**	14.55	0.08	17.75	7.77	8.27	9.68	
**GTS29**	12.73	4.58	19.74	2.61	4.40	8.81	
**GTS77**	3.82	8.69	2.53	21.17	3.71	7.98	
**GTS25**	7.09	11.40	12.85	5.17	3.28	7.96	
**GTS65**	4.52	13.12	4.40	2.47	12.63	7.43	
**GTS31**	10.56	9.07	7.19	5.12	4.92	7.37	
**GTS54**	7.71	0.00	3.06	16.59	0.00	5.47	
**GTS06**	9.34	0.02	3.16	0.00	12.22	4.95	
**GTS73**	4.75	0.63	1.50	9.28	5.34	4.30	

**GTS36**	3.81	1.97	4.15	6.08	4.83	4.17	
**GTS55**	2.43	2.60	2.17	1.19	11.06	3.89	
**GTS28**	0.00	18.02	0.86	0.14	0.14	3.83	
**GTS83**	1.79	1.49	0.76	0.37	12.40	3.36	
**GTS76**	7.70	1.27	1.06	2.31	3.10	3.09	
**GTS82**	3.02	1.74	5.99	0.33	3.79	2.97	
**GTS85**	3.70	1.88	0.79	2.22	0.71	1.86	
**GTS78**	0.31	0.91	0.93	1.21	3.32	1.34	
**GTS07**	0.28	0.14	0.00	0.00	5.97	1.28	
**GTS68**	1.75	1.63	0.25	0.30	0.62	0.91	UDP-glucose
**GTS71**	1.73	0.00	1.06	1.63	0.00	0.88	
**GTS74**	0.00	0.40	2.29	0.00	0.81	0.70	
**GTS10**	508.34	225.79	283.61	426.71	21.16	293.12	
**GTS13**	258.63	127.14	162.74	288.85	21.55	171.78	
**GTS39**	48.56	32.20	77.15	32.16	43.43	46.70	
**GTS26**	29.73	43.68	65.98	20.08	52.54	42.40	
**GTS56**	19.89	29.27	18.90	48.81	25.44	28.46	Shikimate dehydrogenase
**GTS51**	7.33	0.73	11.86	12.49	51.10	16.70	
**GTS44**	9.10	2.52	11.48	6.46	8.97	7.71	
**GTS49**	5.03	0.25	6.50	6.39	13.92	6.42	
**GTS60**	4.44	2.75	3.47	10.60	6.07	5.47	
**GTS33**	0.96	0.93	7.37	3.48	0.82	2.71	
**GTS16**	11.09	11.99	10.27	12.31	14.14	11.96	
**GTS46**	7.55	6.16	5.66	7.38	3.44	6.04	
**GTS19**	1.88	6.18	7.02	4.23	1.82	4.23	
**GTS64**	4.67	2.48	1.09	4.57	1.20	2.80	
**GTS69**	5.64	3.00	1.02	3.01	0.50	2.63	isochorismate synthase synthase
**GTS75**	3.12	2.00	0.57	2.81	0.64	1.83	
**GTS79**	2.89	1.73	0.66	0.65	0.11	1.21	
**GTS84**	0.70	0.80	1.28	0.46	1.13	0.87	
**GTS09**	0.47	1.38	0.00	0.78	0.53	0.63	
**GTS59**	97.03	188.33	63.48	13.93	50.30	82.61	
**GTS24**	10.38	17.57	34.38	20.19	7.02	17.91	chorismate mutase
**GTS37**	7.49	5.92	7.44	3.32	3.69	5.57	
**GTS80**	0.36	1.07	0.36	0.71	0.00	0.50	
**GTS12**	1526.07	2515.44	2447.76	4405.86	2139.91	2607.01	
**GTS40**	138.32	204.95	277.22	83.42	91.40	159.06	
**GTS48**	106.45	109.16	48.06	86.01	28.93	75.72	3-deoxy-d-arabino-heptulosonate-7-phosphate synthase
**GTS34**	48.29	112.16	53.85	45.67	17.94	55.58	
**GTS61**	61.05	87.78	14.44	56.82	11.28	46.27	
**GTS01**	13.96	4.70	4.85	2.96	2.18	5.73	
**GTS20**	126.09	137.69	191.76	333.14	78.90	173.52	
**GTS21**	83.43	124.74	136.90	255.80	54.20	131.01	3-dehydroquinate synthase (DHQ synthase)
**GTS72**	4.64	5.71	2.13	0.00	0.51	2.60	

**GTS62**	0.28	0.81	0.82	0.01	0.87	0.56
**GTS03**	1.82	0.00	0.48	0.00	0.00	0.46
**GTS02**	0.15	0.00	0.30	0.01	0.00	0.09

Table S8. Twenty-two putative genes related to tannin biosynthesis

No.	Gene name	KEGG Orthology	Enzyme	Pathway
**1**	TS-1	K00224	leucoanthocyanidin reductase	flavonoid biosynthesis
**2**	TS-2	K06892	leucoanthocyanidin dioxygenase	
**3**	TS-3	K13082	bifunctional dihydroflavonol 4-reductase/flavanone 4-reductase	
**4**	TS-4	K05277	leucoanthocyanidin dioxygenase	
**5**	TS-5	K13229	leucoanthocyanidin dioxygenase	
**6**	TS-6	K06892	leucoanthocyanidin dioxygenase	
**7**	TS-7	K05279	flavonol 3-O-methyltransferase	flavone and flavonol biosynthesis
**8**	TS-8	K05280	flavonoid 3'-monooxygenase	
**9**	TS-9	K01904	4-coumarate--CoA ligase	
**10**	TS-10	K01904	4-coumarate--CoA ligase	
**11**	TS-11	K01904	4-coumarate--CoA ligase	
**12**	TS-12	K10526	4-coumarate--CoA ligase	
**13**	TS-13	K10526	4-coumarate--CoA ligase	
**14**	TS-14	K13493	anthocyanidin 3-O-glucosyltransferase	anthocyanin and flavanone biosynthesis
**15**	TS-15	K10775	phenylalanine ammonia-lyase	
**16**	TS-16	K13267	flavonoid 6-hydroxylase	isoflavonoid biosynthesis
**17**	TS-17	K13267	flavonoid 6-hydroxylase	
**18**	TS-18	K13258	2-hydroxyisoflavanone dehydratase	
**19**	TS-19	K13258	2-hydroxyisoflavanone dehydratase	
**20**	TS-20	K13258	2-hydroxyisoflavanone dehydratase	

**21**	TS-21	K13258	2-hydroxyisoflavanone dehydratase
**22**	TS-22	K13258	2-hydroxyisoflavanone dehydratase

Table S9. Fifteen putative genes related to plant defense system and hormone biosynthesis

**No.**	**Gene name**	**KEGG Orthology**	**Enzyme**	**Pathway**
**1**	PD-1	K01183	chitinase	plant antibacterial activity
**2**	PD-2	K15563	chitinase	
**3**	PD-3	K01183	chitinase	
**4**	PD-4	K01183	chitinase	
**5**	PD-5	K00517	cytochrome P450	cuticle, tethered layer and wax synthesis
**6**	PD-6	K13407	cytochrome P450	
**7**	PD-7	K11818	cytochrome P450	
**8**	PD-8	K15086	(3S)-linalool synthase	plant terpenoid synthesis
**9**	PD-9	K00511	squalene monooxygenase	
**10**	PHS-1	K00227	lathosterol oxidase	plant hormone synthesis
**11**	PHS-2	K10760	adenylate isopentenyltransferase (cytokinin synthase)	
**12**	PHS-3	K12619	adenylate isopentenyltransferase (cytokinin synthase)	
**13**	PHS-4	K10760	adenylate isopentenyltransferase (cytokinin synthase)	
**14**	PHS-5	K10760	adenylate isopentenyltransferase (cytokinin synthase)	
**15**	PHS-6	K05282	gibberellin 20-oxidase	

Table S10 The quantitative PCR primers of 12 candidate genes in gallnut of *R. chinensis*

Gene ID	Primer sequence	Tm	Gene length
**TS-18**	AATGTTGACTGTTTGGTGAGGG	59	111
	GGAAAGTATTATCGTCTCGTCGTT	59.3	
**TS-19**	GAAATGGACCTGAAACTTGGC	58.8	125
	GACTAATCCGATTGACCCGAC	58.5	
**TS-15**	TAGTTCCTCCTCAAAGGCTCC	57.9	212
	TTCACCCATCAAGGTTCTGC	58.1	
**TS-2**	GGAGGATACAGTGCGGGTTT	58.9	153
	CAATGCGAATTACAAGAGCGT	58.4	
**TS-4**	AAGAATCCCCATTCACGACA	57	181
	TTCCAGAGCGATACATCAAGC	58.5	
**TS-14**	TGTATGCCTTGTTTCACTGACC	57.7	72
	TTGCACCCCAACTTTCCA	57.4	
**TS-8**	AACAGGCATACAGGCGAGAT	57.2	182
	TGCAGCAGGCAAGTTCATAG	57.9	
**TS-9**	AGAGCCTGTTTTCTGTTTCGTC	58.1	164
	TTACTGGGTTCCAAAGTCCGT	58.8	
**PD-1**	CTGTTTTGGATGGGATTGACTT	58.4	137
	AGGAAATGGACACTGAGGAGC	58.2	
**PD-8**	CCCTTCACCCTGTGTTCTAATC	58	219
	TTTTCAGCCAGTGCTACGG	57.1	
**PHS-1**	TGTTGTCAAGAAGGTGGTTTACTAT	57.4	106
	TTTCCATGCTTCTGACATCTGT	57.3	
**PHS-2**	TAACTCAGCCCGTCATTTCTTT	58.7	71
	GTGGGACATATTTGGATTTTGC	58.9	
**Actin**	CATCACTCATCGGTATGGAAGC	59.6	164
	AGTGATTTCCTTGCTCATACGGT	59.9	

Dataset S1. The amino acid sequences translated by the twenty-two putative genes related to condensed tannin biosynthesis in gallnuts of *R. chinensis* after aphids feeding.

TS-1

M G K S K V L V V G A T G Y I G R R I V K A S L A Q G H T T Y V L Q R P E I G L D I E K I Q M L L S F K Q E G A I L V E G S F S D H K S L V D A V K K V D V V I C T M S G *

TS-2

M K I L S L E L G L R E N H L Q N A F G G E N I G A C L R V N F Y P K C P Q P D L T L G L S S H S D P G G L T M L L P D D Q V A G L Q V R K G D N W I T V K P A K H A F I V N I G D Q I Q V L S N A N Y K S V E H R V I V N P E Q E R V S L A F F Y N P K S D I P I E P V K E L V T P D K P A L Y P P M T F D E Y R L F I R L R G P R G K S Q V D S M K S P K C Y *

TS-3

G T L G I L K A C V N S K T V K R V V Y T S S A S A V T F S S N D Q E I V D E I V H G V T *

TS-4

M N K L Q S W P E P I V R V Q S L S E S G L N K I P E R Y I K P S T E R P S F S S E N G I D H H N V N I P I I D L A G L F Q D D Q N V R A Q I I S Q V N M A C R E W G F F Q I L N H G V R P E L M D Q A R E T W R Q F F H A P M E L K Q A Y A N S P K T Y E G Y G S R L G V E K G A I L D W R D Y Y F L *

TS-5

M A A Y D R R K E V E E F D S S K I G V K G L S D S S I T S I P K I F I H P P E T L S T L K S S Q D D T T I T I P V I D L A K I N S P V D R P N I I Q Q V K E A A K S W G F F Q V I N H G I D L S V V E N T I Q A V K S F H E Q P A E M K A K F Y K R E E K S G V M Y A S N N D L Y R S K A A S W H D Y L Q V W M S P E K A K E E D I P E I C R R E A V A W D F H A Q M V G E E V M E L L C E G L G L E A G K F K E L T F S E S R L F V G I Y Y P S C P Q P D L T L G L T P H T D P G A L T V L L Q N Q V P G L Q I K H G D Q W V N V K P L H G S L T I N I G D F L Q I I S N G E Y N S V Q H R V L A N T W E E P R I S V V M F L N V T K W E G S G S H G P L P E L I S A E K P A V Y R K F T K E E Y Y D N F Y S K G L D S K S L I D K L K L *

TS-6

M E A K V L Q K L Q Y P T W V D L D K F Q R K P S G S V R F S R R M R E E K R V Q V L I S A H M Q P S I T S N R N R S V T S E I S C T F Q G F P A S I L E P G G F H A P L D E A L I L K N K S Q E I E P Y L D G R C I Y L V G M M G S G K T T V G K V L S G V L G Y S F F D S D T L I E Q A V G G T T V T D I F K L Y G E G F F R D K E T E V L R K L S I M H R L V V S T G G G A V T R P I N W K Y M R N G I S V F L D V P L E A L A Q R I A A V G T N S R P L L H H E S G D A Y T K A L K R L S T L W E E R S R A Y E N A N A R V C L E N I A A K L G H R D V S A L T P A T I A I E A L E Q I E H F L K E E G E M P F *

TS-7

M E E M K F L K L V V K E T LR L H P P A P L L V P R E C R E K C V I N G F D I P I K T R V I V N A W A I G R D P E Y W S E P E I F N P E

TS-8

L A G F N I S D L F P S I N F L Q W I T K S Q V E K L H Q E A D R I V E K I I N E H K K G Q A P L N I D K S E E A E D L

TS-9

M A A S D S E I V K D F R F F R V Y K D G R V Q L S F P P F V K A T V P P S D D P V T G V Q S K D V T I S S E P P V S A R I F I P K V T D P K Q K F P L L F Y V H G G G F C I M S A F A P G Y H S F C S T I S A Q A G A I V V S V E Y G L F P D R P I P A C Y E D S W A A L Q W V A S H V S G N G P E P C L N D H A D F N K V F I S G V S A G G N I S H T L A F R V G S I G L P G V K V V G V I L V H P Y F G G T G D D E M W L F M C P N N G G L Q D P R L K P P A E D L A K L G C A R V L I F

V A E K D H L N V V G K N Y L E D L K K S G W S G S V E L F E S Y G E G H V F H M L S P E S E K A V E L T N K F V S F L K Q D *

TS-10

M I P R Y I Q N G F F L F I K R I E V T S L S S M A S T D S E I V N E F R F F R V Y K D G R V E L F S P P C P K V P P S A D P V T G V R S K D V T I S S E P L V S A R I F I P K L S D P N Q K F P L L F L V H G G G F C I M S A F A P R Y H L F C N T V S A Q A G A I V V S V E Y G L F P D R P I P A C Y E D S W A A L Q W V A S H V S G N G P E T W L N D H A D F G K V F I G G D S A G G N I S H T L S F R V G S I G L V G V K V V G V I L I H P Y F G G T E D D E M W L Y M N P S N S G L Q D P R L K P P L E D L A K F G C E R V L I F V A E K D H L NT P G K D Y Y E D L K K S G W*

TS-11

M A S S T D S E I A K E F P F F R V Y K D G R V E L F R P H W D K I P P S D D P I T G V R S K D V T I S S E P P V S A R I F I P K L T S P T Q K F P L L L Y V H G G G F S M L S A F S P Q Y H N F C S T V S A L A G V I V V S V E Y G L F P A R P I P A C Y E D S W A A L Q W V A S H V N G N G P D P W L N D H A N F S K V F I G G D S A G G N I S H T L A F R V G S I G L P G M K V D G V I L V H P Y F G G T E D D Q M W L Y M N P T N G G L Q D P R L K P P A E D L G K L G C E R V L I F V A E K D H L Y T P G K D Y H E D L K K S G W G G E V E L V E N H G E E H C F H F H D P K Y E K A V E L T N K F V S F I K Q D *

TS-12

M D S T Q P E I A Y D F S P M L I V Y K D G T V N R L T G N D I V P T S L D P K T N V K S K D V V Y S P E N G L S V R L Y I P K T V S Q N Q K L P L L V Y F H G G G F C I E S P F S P P Y H N Y L N S L V A E A N I V V V S V Q Y R R A P E H H L P C A Y D D S W T A L K W V A S H V N G S G P E D W L N S H A D F E K V F F S G D S A G A N I A H R M G I K H G L E K L E G V N V E G I V L C H P Y F W G N E A V A N E T A D E N R R K F V E A L W R F T Y P E T T G C D D L W V N P A V D P N L A K L G C R R V L V L V A E K D L L C A R G W Y Y H Q K L K E S G W G G D V E V V E T N G E Q H V F H L F N P G C E N A K A M I K K T A D F Y N N

TS-13

M D S S T N E V A H E Y L P Y F R A Y K D G R V E R F F G S D R V P V S L D G S Q N G V C S K D V V I V S E T G L S A R I F I P A G A T K P G Q K L P L V V Y Y H G G G F F M G T P F C S A Y N N S V S S L A A K A N A I A V S V D Y R L A P E H Y V P V A Y E D S W A A L K W V A S H C N G E G P E A W L N N Y A D F H R V F L A G D S A G G N M V H N M A I Q A S V E D L N G V K L S G I C L I Q P Y F G R Q D G G V D K W W T F V C P T T S G S D D H R I N P S V D S R L S S L R C N R V L I C V A E K D N L K E R G L F Y D E T L R D S E W V G E V E I A E T E G E D H V F H L F N P N S E K A V A L L E K I A S F I N Q *

TS-14

L E S I C E G V P M I C M P C F T D Q R V N A R Y V S D V W K V G
V Q L E N G L D R E N I D R T I R R L M V E K E G E E I R G R I L C
L K E K A N I C L R Q G G S S S E S L

TS-15

W R N C K T L N A L L S T L H L F V E T F T E T I S K I G I N Q E G I
T V G L T S F F Q V Q I N V M A W H R A T K K V E D M S F N M S T
A S A V F L E E I R P K E F T S *

TS-16

M N S S A I R Y N E E K E E S F A Y A I Q L V T G S V L P M T L K A
A I D L D I F E I I A K A G P G A K L S A S E I A S R M S A Q N K D A
P M M V D R I L R L L S S H S V L T C Y V V G L E R L Y S L A P V S
K Y F V H D Q N G V S L G P F M A L I Q D K V F M D S W A Q L K D
A V L E G G V P F D R V H G T H A F E Y P G L D A R F N Q V F N T A
M F N H T T I V I N E I L K V Y K G F E S I K Q L V D V G G G L G I T
L K A I T S R Y P H V T A I N F D L P H V I Q H A P Q N P G V E H V
G G D M F E S V P K G D A I F M K W I L H D W S D E H C L K L L K
N C Y K S I P E D G K V I V V E G I L P V M A E T K V T S K G V S Q
M D V V M M T Q N P G G K E R T K H E F M A L A T A A G F S G I R
Y E C F V C N F W V M E F Y K *

TS-17

M E A F S W V V L A T A W L A A L V F L K R I S T S W R Q S L N S I
P G P K P W P I I G N L N L I G P L P H Q S L H K L A Q K Y G S I M Q
L K F G S F P V V V A S S A E M A K L F L R T H D H I F A S R P P T
A A G K Y T T Y N Y L N I T W A P Y G P Y W R Q G R K M Y L A E L
F S S K R L E S Y E Y I R V E E L R D S M S R L Y A L S G K P I V L K
D H L S R F T L S I I S R I V L G K K Y F C V S K S E T S V V T L E E
F Q E M M D E L F L L N G V I N I G D W I P W M D F L D L Q G Y V
K R M K V L K K R L D R F H D H V F D E H K S K R E G V K D F E A
K D M V D L L L R L A D D P N F D A K L T S D S I K G F T Q D L I A
G G T D T A A T T V E W A M S E

TS-18

M A A G N D I D D L P K N A A N Y T A L T P L W F L E R A A T V H P
S R A S L I H G S V R Y T W H Q T Y Q R C R R F A S A L S R R S V G
L G S T V A V I A P N V P A L Y E A H F G V P M A G A V L N S V N I
R L N A Q T I A F L L G H S A S A V V M V D Q E S F S V A E E A L K
I M E E K S K G N F K P P I L I V I G D E S C D P K V L E Y A L G R G
A I E Y E K F L E T G D P E F A W K P P Q D E W Q S I A L G Y T S G
T T A S P K G V V L S H R G A Y V M S M S G A L I W G L N E G A V
Y L W T L P M F H C N G W C Y T W S L A A L C G T N I C L R Q V T
A K A V Y S A I A K Y G V T H F C A A P V V L N T I V N A S P E D T
I L P L P H V V N V M T A G A A P P Q S V L F S M S Q K G F R V T H
T Y G L S E T Y G P S T V C A W K P E W N S L P P E T Q A R L N S R
Q G V R Y I G L E F L D V I D T R T K K P V P A D G K T I G E I V L G

G N S V M K G Y L K N P K A N E E A F A D G W F H S G D L G V K H
P D N Y I E I K D R S K D I I I S G G E N I S S L E V E N L L Y L H P
A I L E A S V V A R P D E R W G E S P C A F I T L K Q G A D K D E P
R L A E D I M K F C R E K M P A Y W V P K S V I F G P L P K T A T G
K I Q K H I L R A K A K E M G P V K R S K L *
TS-19
M E V Q A K P Q E E L I I Y R S K L P D I Y I P N H L P L H S Y C F E
N I A Q V A S R P C L I N G S T G D V Y T Y S D V E L T A R K I A S
G L N K L G I Q Q Y D V I M L L L P N F P E F I L S F L G A S Y R G A
I V T A A N P L C T P A E I S K Q A E A S N A K L I I T Q A C Y V D K
V K A L S E K R D V K I M C I D A A P D G C L H F S E L T Q A D E N
D L P E V K F N P D D V V A L P Y S S G T T G L P K G V M L T H K G
L V T S V A Q Q V D G E N P N L Y F H S E D V I L C V L P L F H I Y
A L N S I F L C G L R A G A S I L L M Q R F E I N S L L Q L I Q R Y K
V T V A P M V P P I V L A I A K S P D V D K H D M S S I R F I K S G A
A P L G K E L E D S V R A K F P N A T L G Q G Y G M T E A G P V L A
M G L A F A K H P F P I K P G A C G T V V R N A E M K I I D P D T G
A Y L P R N Q P G E I C I R G D Q I M K G Y L N D Q E A T K R T I D
S D G W L H T
G D I G Y I D D D D E L F I V D R L K E L I K Y K G F Q V A P A E L
E A M L L T H S S I S D A A V V P M K D D Q A G E V P V A F V V K A
K D S Q I T E D E V K Q F I S K Q V V F Y K R I N R V F F I D A I P K
A P S G K I L R K D L R A K L A A G L P N *
TS-20
M E G T I R C S A N Y V P L S P I S F L E R S A I V Y R D R L S V V Y
E D V K Y T W K E T H Q R C I K L A S A L A D L G I S R G D V V A A
L A P N I P A M Y E L H F G V P M A G A V L C T L N V R H D S A M
V A V L L K H S E A K I I F V D H Q L L D T A R A A V E I L A K S T T
K L P L L V I I P E C S T V R N P I P A H E N L E Y E S L L A K G I L
D F E V R R P K D E W D P I S L N Y T S G T T S S P K G V I Y S H R
G A Y L N S L A V A L L N E M S S M P T Y L W C V P M F H C N G W
C L T W A V A A Q G G T N V C L R I V N A K G I F D N V A R Y K V
T H L G G A P T V L N M I V N A A A G E R R P L P G K V V V M T G
G A P P P P Q V L F S M E E L G F V V T H S Y G L T E T Y G P G T V
C T W K P E W D S L P R E A Q A K I K A R Q G L H H L G M E E V D
I K D P V T M K S V P P D A K T M G E A M F R G N T V M N G Y L K
N S K A T Q D A F N G G W Y R
S G D L G V K H P D G Y I E L K D R S K D I I I S G G E N I S T I E V
E S V L F S H P S V L E A A V V G R P D D H W G E T P C A F V K L K
D G C D A S S E E L I K Y C R S R L P H F M A P R T I V F Q D L P K
T S T G K T Q K F V L R E K A K A M G S I S K Q R S S K L *
TS-21
M E G T I R C S A N Y V P L S P I S F L E R S A I V Y R D R L S V V Y
E D V K Y T W K E T H Q R C I K L A S A L A D L G I S R G D V V A A

L A P N I P A M Y E L H F G V P M A G A V L C T L N V R H D S A M
V A V L L K H S E A K I I F V D H Q L L D T A R A A V E I L A K S T T
K L P L L V I I P E C S T V R N P I P A H E N L E Y E S L L A K G I L
D F E V R R P K D E W D P I S L N Y T S G T T S S P K G V I Y S H R
G A Y L N S L A V A L L N E M S S M P T Y L W C V P M F H C N G W
C L T W A V A A Q G G T N V C L R I V N A K G I F D N V A R Y K V
T H L G G A P T V L N M I V N A A A G E R R P L P G K V V V M T G
G A P P P P Q V L F S M E E L G F V V T H S Y G L T E T Y G P G T V
C T W K P E W D S L P R E A Q A K I K A R Q G L H H L G M E E V D
I K D P V T M K S V P P D A K T M G E A M F R G N T V M N G Y L K
N S K A T Q D A F N G G W Y R
S G D L G V K H P D G Y I E L K D R S K D I I I S G G E N I S T I E V
E S V L F S H P S V L E A A V V G R P D D H W G E T P C A F V K L K
D G C D A S S E E L I K Y C R S R L P H F M A P R T I V F Q D L P K
T S T G K T Q K F V L R E K A K A M G S I S K Q R S S K L *

TS-22

M E Q R S L H T D P K S G F N S L T K T F H S L K A P V H L P P E N
A F L S A A E Y A F S L R A T S P W P D D S V A L V N S I T G Q R I S
Y S E F G H R T K S L A A Y L Q K V T Q L S K H D V A F V L S P N S
I Q V P I L Y F S L L S L G V I I S P A N P V A T E S E I Y G Q I Q L S
K P V I A F A R S S T V H K L P K L K H Q T I L I D S P E F E S M M V
S S K H E F K H V K L S Q S D P A A I M Y S S G T T G K V K G V M L
T H R N F I A Q T A I N S A A W K M S E K R E S P S A M L F T T P Y
F H I F G F F Y S I R S V A L S E K A V V M D R F D W K N M L K A V
Q E F S V T H V A L T P P V V V K L S K D G S T D D Y D L S S L E T
V I C G A A P L G K E A I A A F T S R F P K V M L V Q A Y G L T E S
T A A V A R T I G L E E N M K W G S T G K L Y A G F E A K V V D P
E T S D A L P P C K E G E L W I R G P T I M K G Y V G D P E A T S A
T L V C D G W M R
T G D L C Y I D E N G F L F I V D R L K E L I K Y K G Y Q V A P A E
L E Q V L I S H P Q V A D A A V I P Y P D K E T G Q V P M A F V V R
Q P Q S T L N E T E I M D F V A K Q V A P Y K K I R R V A F I N S I P
K S A A G K I L R K D L I R K F V L P P P A S S R L *
